# Inhibition of Heme Oxygenase-1 by Zinc Protoporphyrin IX Improves Adverse Pregnancy Outcomes in Malaria During Early Gestation

**DOI:** 10.3389/fimmu.2022.879158

**Published:** 2022-05-10

**Authors:** Yusmaris Cariaco, Marcos Paulo Oliveira Almeida, Ester Cristina Borges Araujo, Marisol Patricia Pallete Briceño, Andrea Tatiana Durán-Rodriguez, Rodrigo Rodrigues Franco, Foued Salmen Espindola, Neide Maria Silva

**Affiliations:** ^1^Laboratory of Immunopathology, Institute of Biomedical Sciences, Federal University of Uberlândia, Uberlândia, Brazil; ^2^Laboratory of Biochemistry and Molecular Biology, Institute of Biotechnology, Federal University of Uberlândia, Uberlândia, Brazil

**Keywords:** HO-1, malaria, pregnancy, uterine natural killer cells, iron overload, ferroptosis, ZnPPIX.

## Abstract

The enzyme heme oxygenase-1 (HO-1) has cytoprotective effects by catalyzing the degradation of heme to produce carbon monoxide, iron and biliverdin. Furthermore, HO-1 activity has been associated with successful pregnancy. On the other hand, in the context of certain inflammatory conditions, HO-1 can induce iron overload and cell death. To investigate the role of HO-1 in gestational malaria, pregnant BALB/c mice were infected with *Plasmodium berghei* ANKA in early, mid and late gestation. We found that malaria affected the pregnancy outcome in the three periods evaluated. However, only poor pregnancy outcomes in early pregnancy were related to HO-1 upregulation, iron overload, lipid peroxidation and necrosis of the decidua, which were prevented by HO-1 inhibition. In conclusion, HO-1 expression must be finely tuned in gestational malaria to avoid the deleterious effect of increased enzyme activity.

## Introduction

Malaria in pregnancy is a serious public health problem. According to the World Health Organization, the prevalence of exposure to malaria during pregnancy was 29% in areas of moderate to high transmission in sub-Saharan Africa in 2018, where more than 11 million pregnant women acquired malaria in that year, and approximately 872,000 children were born with low birthweight due to the infection. These children are at high risk of stunted growth, poor cognitive development and death (1). Malaria in pregnancy can induce different outcomes for the mother and her offspring, potentially causing maternal anemia, severe malaria and maternal mortality. In addition, if infection occurs between the first and second trimester of pregnancy this may result in early abortion, intrauterine growth restriction and low birthweight (2).

Malaria is a hemolytic disease caused by protozoan parasites of the genus *Plasmodium*, in which heme released during the rupture of red blood cells leads to oxidative damage due to the production of reactive oxygen species that induce inflammation and tissue damage (3, 4). Because heme is also toxic to parasites, they turn heme into a non-toxic product, the malarial pigment known as hemozoin (5). However, the main mechanism of catalysis of heme is through the activity of the enzyme heme oxygenase 1 (HO-1), which is a rate-limiting enzyme that catalyzes the degradation of heme to produce carbon monoxide (CO), iron and biliverdin (BV). The generated biliverdin is later converted to bilirubin by biliverdin reductase (6, 7). HO-1 exerts cytoprotective effects through the removal of heme and by generating degradation products such as bilirubin and CO, which have antioxidant, vasodilator, anti-inflammatory and anti-apoptotic properties (8, 9) which are also important at the feto-maternal interface (10–12).

Despite the beneficial aspects frequently associated with HO-1 activity, excessive formation of degradation products as a result of increased expression can also lead to adverse effects. Iron overload and cell death have been associated with increased HO-1 activity in some inflammatory contexts such as *Mycobacterium tuberculosis* infection (13, 14), neuroinflammation during aging (15) and septic shock (16). The detrimental effects of the elevated expression of HO-1 are commonly attributed to iron overload due to its pro-oxidant nature, suggesting that the expression of this enzyme requires delicate modulation to preserve its beneficial effects (8). Labile iron is one of the main causes of lipid peroxidation, modifying physical properties of cell membranes, leading to loss of membrane permeability and cell death (17). In this sense, iron overload and the resulting lipid peroxidation may cause ferroptosis, a regulated non-apoptotic form of cell death marked by loss of activity of the lipid repair enzyme glutathione peroxidase 4 (GPX4) (18). GPX4 is the only enzyme capable of reducing hydroperoxides within membranes, making it a ferroptosis regulator molecule (19).

In mice, embryo implantation takes place when the blastocyst attaches to the cells of the luminal epithelium of the endometrium, inducing the process of decidualization, through which uterine stromal cells proliferate and differentiate into decidual cells (20). Simultaneously with decidualization, a highly organized lymphoid structure is formed adjacent to the decidua, the mesometrial lymphoid aggregate of pregnancy (MLAp) (21). Uterine natural killer cells (uNK) are abundant in decidual tissue and MLAp, constituting approximately 70% of the leukocytes infiltrating the decidua, where they fulfill important functions for the maintenance of pregnancy, among them the promotion of spiral artery (SA) remodeling (22, 23). Decidual cells supply nutritional support to the developing embryo until the establishment of the definitive placenta, which has a maternal layer (the decidua) and a fetal part formed by the junctional zone (which contains several trophoblast cells types), below which is the labyrinth layer, where gas and nutrient exchanges between mother and fetus occur (20, 24). HO-1 expression has been shown to be important for fetal tolerance, maintenance of placental architecture, uNK cell differentiation, SA remodeling, and preservation of normal blood pressure during pregnancy (25–28).

Previous studies have shown that experimental infection by abortion-causing bacteria such as *Listeria monocytogenes* and *Brucella abortus* leads to the reduced expression of HO-1 in trophoblast giant cells of the placenta, and the pharmacological induction of the enzyme prevented infectious abortion (29, 30). On the other hand, it has been reported that *Plasmodium berghei* ANKA infection induces an increase in protein and mRNA expression of HO-1 in the brain of BALB/c mice and in the lungs of DBA mice, conferring protection against the onset of experimental cerebral malaria and acute lung injury, respectively (31, 32). Interestingly, a previous report showed that iron overload in trophoblast cells in placentas from mice infected with *Plasmodium berghei* was associated with fetal death (33). However, the exact role of HO-1 and the iron status at the feto-maternal interface throughout pregnancy remains unclear in the context of malaria infection.

Here, we conduct experiments to determine the impact of *Plasmodium* infection and HO-1 modulation on pregnancy outcomes during malaria using a mouse model of malaria in pregnancy, which reproduces most of the pathophysiological features observed in human pregnancy-associated malaria. Thus, three time points of murine pregnancy, equivalent to time points within the three trimesters of human gestation, were evaluated to study essential processes for the success of pregnancy, such as decidualization, remodeling of spiral arteries, placentation and fetal growth.

## Results

### *Plasmodium* Infection in Early Pregnancy Leads to a Decrease in uNK Cells and Embryonic Loss

**Figure 1:**
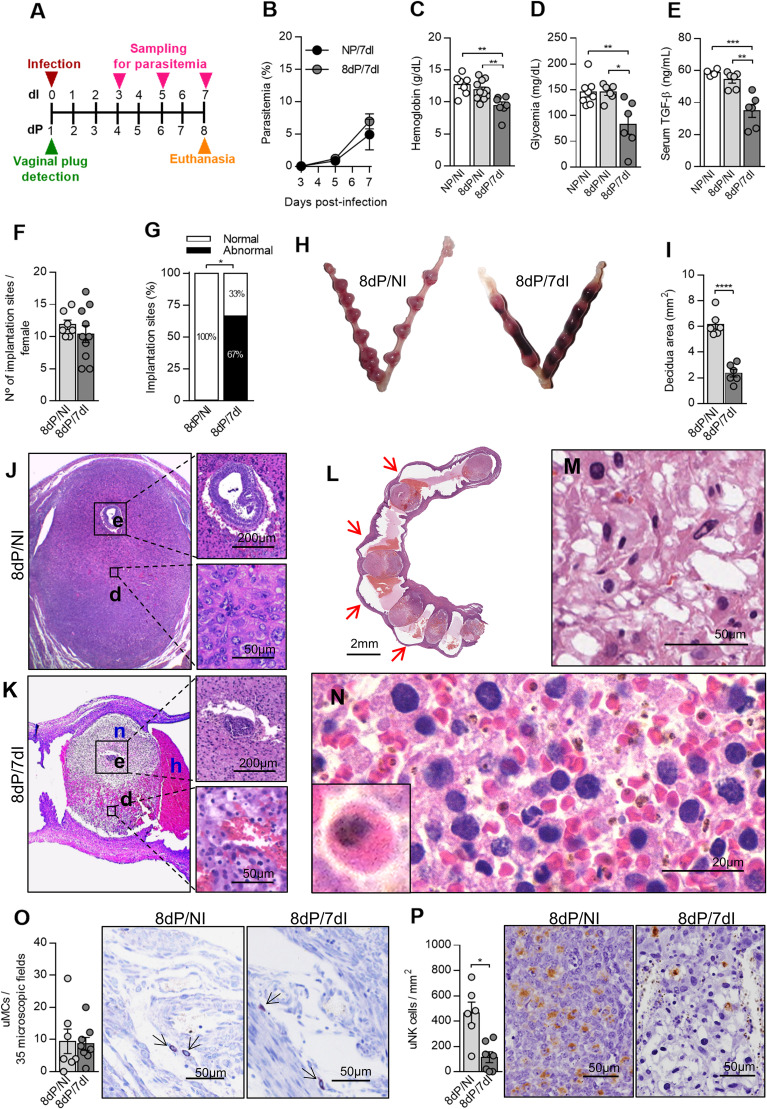
*Plasmodium berghei* ANKA infection causes poor pregnancy outcome during early pregnancy. **(A)** An experimental design for the study of malaria in early gestation using BALB/c mice infected with *P. berghei* ANKA was performed. **(B)** Parasitemia levels were estimated by flow cytometry (n = 5 mice/group). **(C–E)** Hemoglobin (NP/NI, n=7; 8dP/NI, n=11; 8dP/7dI n=7), blood glucose (NP/NI, n=8; 8dP/NI, n=6; 8dP/7dI, n=6) and serum TGF-β levels were measured (NP/NI, n=4; 8dP/NI, n=6; 8dP/7dI, n=6). **(F–H)** The number of implantations sites per female was counted (8dP/NI, n=7; 8dP/7dI, n=10) **(F)** and classified as normal or abnormal **(G)** according to macroscopic aspect **(H)** (8dP/NI, n=7; 8dP/7dI, n=10). **(I-N)** Uterine tissue was histologically processed and tissue sections were stained by H&E. Decidual area was delimited **(I)** and implantation sites of uninfected **(J)** and infected mice **(K) **were evaluated. e, embryo; d, decidua; n, necrosis; h, hemorrhage. Most of the infected mice presented intrauterine hemorrhages, which are indicated by red arrows **(L)**, decidual edema **(M)** and decidual hemorrhage, where infected red blood cells were frequently observed **(N). (O)** uMCs were detected and quantified in Toluidine Blue stained implantation sites (8dP/NI, n=7; 8dP/7dI, n=8). **(P)** uNK cells were detected and quantified in DBA lectin-stained implantation sites (8dP/NI, n=6; 8dP/7dI, n=7). Results represent mean ± SEM. Data was analyzed by Two-way ANOVA followed by Bonferroni’s post-test **(B)**, One-way ANOVA with Bonferroni’s *post-hoc* comparisons tests **(C–E)**, unpaired t-test **(F, I, O, P)** or Mann-Whitney test **(G)**. NP, non-pregnant, NI, non-infected, dP, days of pregnancy, dI, days of infection. *: p <0.05; **: p <0.01; ***: p <0.001; ****: p <0.0001.

In order to verify the decidualization and embryo development in early pregnancy and to describe some of the alterations induced by infection during this stage, we inoculated mice with 5×10^4^ infected red blood cells by intraperitoneal route in the first day of pregnancy (dP) and euthanized them 7 days later (**Figure 1A**). We observed that pregnant infected mice presented parasitemia levels similar to those of non-pregnant infected mice (NP/7dI) (**Figure 1B**). Since anemia, hypoglycemia and lower serum levels of TGF-β are common alterations during malaria [reviewed by (34–36)] and have been observed in pregnant women with malaria (1, 37–40), these parameters were measured in the animals in this experimental model. We noted that mice infected during early pregnancy displayed lower levels of hemoglobin (p<0.0068), glycemia (p<0.0101) and serum TFG-β (p<0.0011) than non-pregnant uninfected (NP/NI) and pregnant uninfected mice (8dP/NI) (**Figures 1C–E**).

We next evaluated the impact of infection on pregnancy outcome. It was shown that infection did not affect the number of embryos implanted in the uterine horns of each female (**Figure 1F**), even the parasites were inoculated before implantation, the latter estimated to occur 4–5 days after vaginal plug detection (41), which coincides with the rising of parasitemia levels on day 5 after infection (**Figures 1A, B**). Despite this observation, we detected a significantly reduced proportion of normal implantation sites (33%) in infected pregnant females when compared with non-infected pregnant counterparts (100%) (p=0.0278) in which no abnormal implantation sites were detected (**Figures 1G, H**). We then performed histological evaluations of the implantation sites and found that the infected females had implantation sites with a marked reduction in the size of the decidual tissue (p<0.0001) (**Figures 1I–K**).

Decidual cell morphology on the mesometrial side of the uterus of pregnant uninfected females was consistent with a previous description (42), these cells being large, with oval nuclei, several punctate nucleoli and pale eosinophilic cytoplasm (**Figure 1J**). In contrast, we observed that mesometrial decidua in the uterus of pregnant infected females showed edema (**Figure 1M**), extensive hemorrhagic areas full of parasitized red blood cells (**Figure 1N**) and decidual cells with pyknotic nuclei (**Figure 1K**). At the same time, while uninfected females displayed normal embryonic development for the gestational age (**Figure 1J**), infected females presented necrosis of the entire decidua including the embryo (**Figure 1K**). Moreover, vaginal bleeding and intrauterine hemorrhage (**Figures 1H, L**) between implantation sites of infected females was often observed at the time of euthanasia.

Chymases secreted by uterine mast cells (uMCs) and uNK cells are essential for the vascular changes required to support pregnancy (43). It has been suggested that the effects induced by a deficiency in one of these cell types are counterbalanced by the other, promoting spiral artery remodeling and placentation (44). In order to verify whether infection with *Plasmodium* affects these cells in early pregnancy, we quantified these cell phenotypes using histochemical techniques. We observed that although uMC numbers were not affected (**Figure 1O**), uNK cells were significantly reduced in infected pregnant animals in relation to non-infected pregnant females (p=0.0303) (**Figure 1P**). Thus, *Plasmodium* infection leads to significant alterations in both mother and developing embryo, leading to pregnancy loss associated with a reduction in uNK cells numbers when mothers are infected in early pregnancy.

### *Plasmodium* Infection Impairs the Pregnancy Outcome During Mid-Gestation Without Altering uNK Cell Numbers

**Figure 2:**
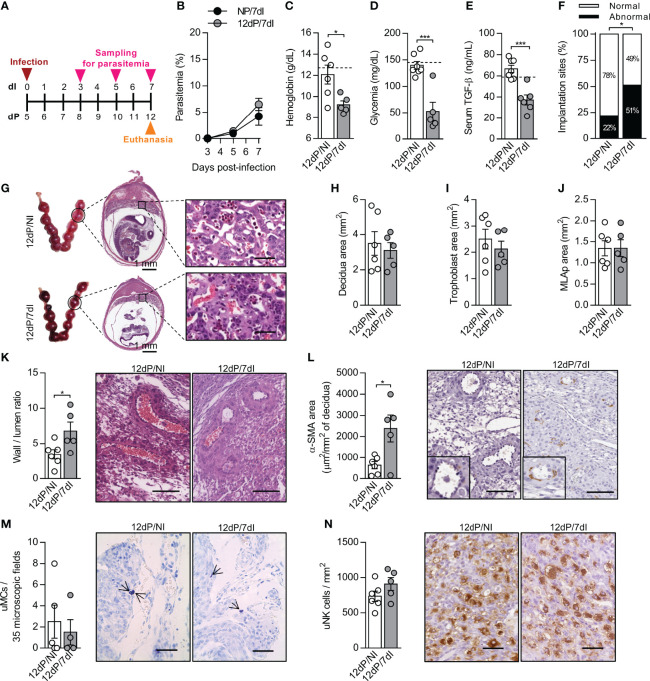
Malaria interferes in the remodeling of spiral arteries without changes in uNK cells numbers in mid-pregnancy. **(A)** An experimental design for the study of malaria in mid-gestation using BALB/c mice infected with *P. berghei* ANKA was performed. **(B)** Parasitemia levels were estimated by flow cytometry (n = 6-7 mice/group). **(C–E)** Hemoglobin (12dP/NI, n=6; 12dP/7dI, n=5), blood glucose (12dP/NI, n=7; 12dP/7dI, n=5) and serum TGF-β levels were measured (12dP/NI, n=7; 12dP/7dI, n=6). The dashed lines indicate the mean results of non-pregnant uninfected mice for each parameter evaluated. **(F, G)** The implantations sites were classified as normal or abnormal **(F)** according to macroscopic aspect (12dP/NI, n=7; 12dP/7dI, n=6), histologically processed and stained by H&E **(G)**, scale bar 50 μm. **(H–J)** Decidua **(H)**, trophoblast **(I)** and MLAp **(J)** areas were delimited and quantified (12dP/NI, n=6; 12dP/7dI, n=5). **(K)** Wall-to-lumen area ratio was evaluated in the decidua of implantation sites stained by H&E (12dP/NI, n=6; 12dP/7dI, n=5), scale bar 100 μm. **(L)** α-SMA staining was quantified in decidual tissue for assessment of spiral arteries remodeling (12dP/NI, n=6; 12dP/7dI, n=5), scale bar 100 μm. **(M)** uMCs were detected and quantified in Toluidine Blue stained implantation sites (12dP/NI, n=5; 12dP/7dI, n=4), scale bar 50 μm. **(N)** uNK cells were detected and quantified in the decidua of DBA lectin-stained implantation sites (12dP/NI, n=6; 12dP/7dI, n=5), scale bar 50 μm. Results represent mean ± SEM. Data was analyzed by Two-way ANOVA followed by Bonferroni’s *post-hoc* comparisons tests **(B)** and unpaired t-test **(C–F, H–N)**. NP, non-pregnant, NI, non-infected, dP, days of pregnancy, dI, days of infection. *: p <0.05; ***: p <0.001.

In order to determine the impact of *Plasmodium* infection in mid-pregnancy, mice were infected on day 5 of pregnancy and euthanized 7 days later (**Figure 2A**). As in early pregnancy (**Figure 1B**), parasitemia levels were similar between pregnant and non-pregnant animals (**Figure 2B**). Additionally, infected pregnant mice displayed lower levels of hemoglobin (p=0.0233), blood glucose (p=0.0004) and serum TGF-β (p=0.0003) in relation to uninfected pregnant controls (**Figures 2C–E**). When evaluating the pregnancy outcome, it was observed that non-infected mice presented a small number of abnormal implantation sites, whereas on the other hand, infected mice exhibited twice as many abnormal implantation sites as uninfected females (p=0.0430) (**Figures 2F, G**). Additionally, it was observed that vascular spaces in the placenta labyrinth layer of infected mice were reduced due to the thickening and disorganization of the trophoblast barrier. Simultaneously, infected red blood cells were detected in maternal blood vessels of placentas (**Figure 2G**).

Crucial changes take place in uterine vasculature during mid-gestation. Artery remodeling is a process that renders the spiral arteries into low-resistance, high-capacity vessels, allowing the correct flow of blood to reach the developing fetus (45, 46). Meanwhile, each placental layer fulfills important functions for the maintenance of pregnancy, with the suggestion that inappropriate proportions of the placental layers and poor remodeling of the spiral arteries can lead to intrauterine growth restriction and even embryo loss (47). Although we did not detect significant changes in the size of the MLAp, decidua and trophoblast layers in response to infection (**Figures 2H–J**), poor remodeling of the spiral arteries in infected mice during mid-gestation was observed, as indicated by an increased wall-to-lumen ratio (p=0.0378) (**Figure 2K**), which means that the arteries had a thicker wall and smaller lumen. In accordance, the α-smooth muscle actin (α-SMA) staining was increased (p=0.0177) in deciduas of infected animals, revealing a higher proportion of smooth muscle cells in spiral arteries (**Figure 2L**), a well-known feature of unremodeled arteries. Unexpectedly, the numbers of uNK cells and uMCs, two cell populations associated with the promotion of SA remodeling, were unchanged after infection (**Figures 2M, N**). We speculate that even in the presence of a similar number of uNK and uMCs cells, they could be differentially expressing molecules that assist in arterial remodeling, as observed in previous studies (48, 49).

### *Plasmodium* Infection in Late Gestation Impairs Fetal Growth

**Figure 3:**
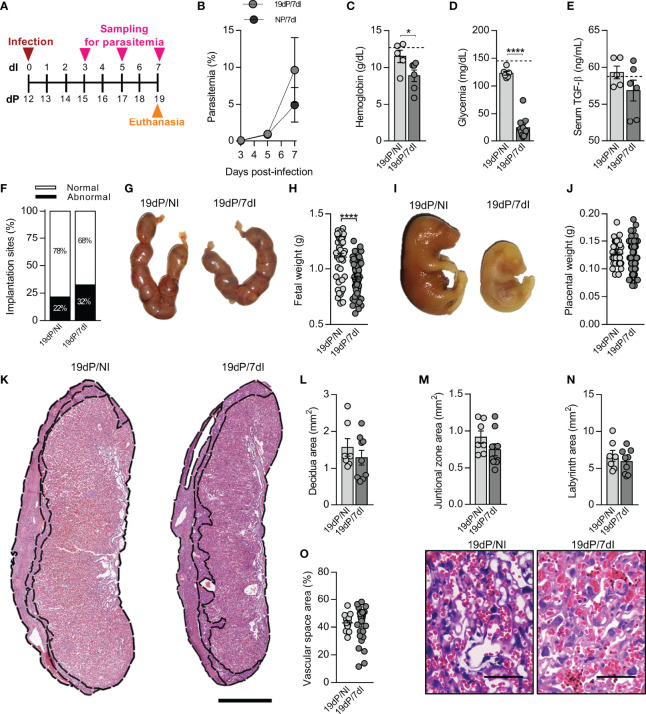
Malaria impairs maternal and fetal outcomes in late pregnancy. **(A)** An experimental design for the study of malaria in late gestation using BALB/c mice infected with *P. berghei* ANKA was performed. **(B)** Parasitemia levels were estimated by flow cytometry (n = 5 mice/group). **(C–E)** Hemoglobin (19dP/NI, n=5; 19dP/7dI, n=6), blood glucose (19dP/NI, n=6; 19dP/7dI, n=11) and serum TGF-β levels (19dP/NI, n=5; 19dP/7dI, n=6) were measured. The dashed lines indicate the mean results of non-pregnant uninfected mice for each parameter evaluated. **(F, G)** The implantations sites were classified as normal or abnormal **(F)** according to macroscopic aspect (19dP/NI, n=7; 19dP/7dI, n=11) **(G)**. **(H–J)** Fetal and placental weight were recorded. Each point represents the measurement of a placenta or fetus (19dP/NI, n=42 fetuses/placentas derived from 7 litters; 19dP/7dI, n= 77 fetuses/placentas derived from 11 litters). **(K–N)** Placental sections were stained by H&E **(K)** for delimitation of decidua **(L)**, junctional zone **(M)** and labyrinth **(N)** layers (19dP/NI, n=7; 19dP/7dI, n=9), scale bar 1 mm. **(O)** Vascular space area in labyrinth layer was estimated in placenta sections stained with H&E (19dP/NI, n=16 placentas derived from 7 litters; 19dP/7dI, n=29 placentas derived from 11 litters), scale bar 50 μm. Results represent mean ± SEM. Data was analyzed by Two-way ANOVA followed by Bonferroni’s *post-hoc* comparisons tests **(B)**, unpaired t-test **(C–F, J, M–N)** and Mann-Whitney test **(H, L, O)**. NP, non-pregnant, NI, non-infected, dP, days of pregnancy, dI, days of infection. *: p <0.05; ****: p <0.0001.

Most of the pregnancy-associated malaria complications in both human and mice have been reported to take place in late pregnancy (2, 50–52). To verify whether the maternal and fetal parameters were altered after infection in our experimental model in late pregnancy, mice were infected on 12dP and euthanized 7 days later on 19dP (**Figure 3A**). It was observed that parasitemia levels in pregnant infected were similar to those in infected non-pregnant mice (**Figure 3B**). On the other hand, infected pregnant mice exhibited lower hemoglobin (p=0.0181) and glycemia levels (p= 0.0022) than uninfected pregnant females (**Figures 3C, D**), but unlike the previous gestational periods, no change in TGF-β serum levels was observed in response to infection (**Figure 3E**).

When examining the ratio of normal and abnormal implantation sites, no differences were found between infected and uninfected animals (**Figures 3F, G**). Nevertheless, infection caused a significant decrease in fetal weight (p<0.0001) (**Figures 3H, I**) and size (**Table 1**), without affecting placental weight (**Figure 3J**), the size of placental layers (**Figures 3K–N**) or the vascular space in the labyrinth layer of the placenta (**Figure 3O**).

**Table 1:** Fetal biometry measurements in fetuses of BALB/c mice infected or not with *P. berghei* ANKA.

Parameter	19dP/NI	19dP/7dI	p-value
CRL (cm)			
Mean ± SEM	2.234 ± 0.0134	2.053 ± 0.0285	<0.0001
Minimum	1.943	1.488	
Maximum	2.481	2.437	
SOD (cm)			
Mean ± SEM	1.104 ± 0.0069	1.048 ± 0.0096	<0.0001
Minimum	0.950	0.885	
Maximum	1.201	1.196	
APD (cm)			
Mean ± SEM	0.927 ± 0.0128	0.876 ± 0.0112	0.0080
Minimum	0.846	0.690	
Maximum	1.031	1.053	
BPD (cm)			
Mean ± SEM	0.760 ± 0.0136	0.733 ± 0.0091	0.1204
Minimum	0.687	0.589	
Maximum	0.878	0.853	

CRL, Crown-rump length, SOD, snout-occipital distance, APD, abdominal anteroposterior diameter, BPD, biparietal diameter, SEM, standard error of the mean. 19dP/NI group (n= 8 litters, 62 pups); 19dP/7dI group (n=5 litters, 46 pups). The differences between the groups were determined by unpaired t-test. p-values <0.05 were considered significant.

### HO-1 Is Upregulated During Malaria in Early Pregnancy Inducing Iron Overload at Implantation Sites

**Figure 4:**
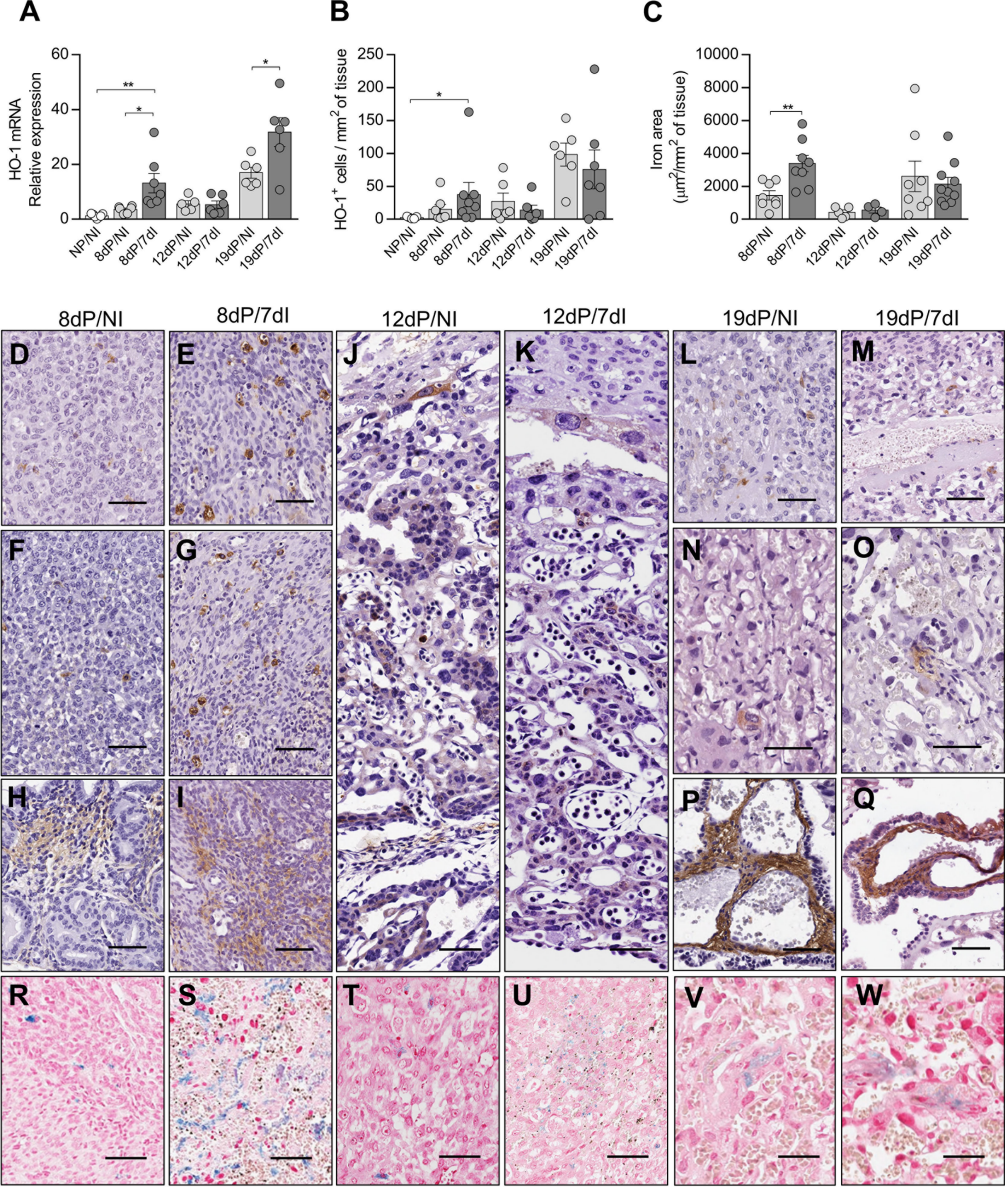
Expression of HO-1 is increased in early pregnancy leading to iron overload in implantation sites. **(A)** mRNA expression of HO-1 in uterus/placenta was measured by qPCR normalizing to GAPDH expression and using NP/NI group as calibrator (NP/NI, n=7; 8dP/NI, n=6; 8dP/7dI, n=7; 12dP/NI, n=5; 12dP/7dI, n=6; 19dP/NI, n=6; 19dP/7dI, n=6). **(B)** The number of HO-1^+^ cells in uterus/placenta were detected by immunohistochemistry and normalized according to the tissue area evaluated (NP/NI, n=5; 8dP/NI, n=7; 8dP/7dI, n=8; 12dP/NI, n=6; 12dP/7dI, n=6; 19dP/NI, n=6; 19dP/7dI, n=7). **(C)** Iron-stained area was quantified in tissue sections of uterus/placenta stained by Perls’ method and normalized according to the tissue area evaluated (8dP/NI, n=8; 8dP/7dI, n=8; 12dP/NI, n=5; 12dP/7dI, n=4; 19dP/NI, n=8; 19dP/7dI, n=10). **(D–Q)** Representative images of HO-1-stained tissue, showing protein expression in decidual tissue **(D–G, L,M)**; endometrium **(H, I)**; trophoblast cells of mid-gestation placenta **(J, K)**; and labyrinth region **(N, O)** and chorionic plate **(P, Q)** of late gestation placenta, scale bar 50 μm. **(R–W)** Representative images of iron-stained tissue, showing positive staining at decidua **(R–U)** and labyrinth region of the placenta **(V, W)**. Each symbol represents the measure of one mouse and results represent mean ± SEM. Data was analyzed by unpaired test-t. NP, non-pregnant, NI, non-infected, dP, days of pregnancy, dI, days of infection. *: p <0.05; **: p <0.01.

HO-1 activity has protective effects during pregnancy (11, 12, 25, 53). In parallel, *Plasmodium* infection is characterized by the release of large amounts of heme (54). However, there is a lack of studies about the role of HO-1 in the pregnancy outcome during malaria. Thus, we measured the gene and protein expression of this enzyme in the uterus/placenta of infected mice during early, mid and late gestation. In early pregnancy, HO-1 mRNA expression levels were increased in the uterine tissue of pregnant infected mice in relation to uninfected non-pregnant (p=0.0018) and uninfected pregnant mice (p=0.0129) (**Figure 4A**) and the quantification of HO-1^+^ cells by immunohistochemistry showed that infected pregnant mice had a greater number of positive cells in relation to non-pregnant uninfected mice (p=0.0375) (**Figure 4B**). Moreover, the upregulation of HO-1 was associated with an increased iron deposition at the implantation sites of infected mice when compared with uninfected pregnant females (p=0.0137) (**Figures 4C, R, S**). HO-1 protein detection by immunohistochemistry revealed that staining was mainly found throughout the placenta (**Figures 4J, K, N, O**), in scattered cells in the decidua (**Figures 4D–G, L, M**), in stromal cells in the endometrium (**Figures 4H, I**) and in the chorionic plate (**Figures 4P, Q**).

Unlike in early pregnancy, during mid-pregnancy infection did not affect the gene (**Figure 4A**) or protein expression of HO-1 (**Figures 4B, J, K**), nor did it lead to iron accumulation at the implantation sites (**Figures 4C, T, U**). In late pregnancy, despite a significant increase in the mRNA expression of HO-1 in the infected group (p=0.0004) (**Figure 4A**), there was no increase in the number of HO-1^+^ cells or in iron deposition in the placentas (**Figures 4B, C, V, W**). Therefore, our results suggest that HO-1 plays a role in the pathogenesis of malaria during early pregnancy.

### Inhibition of HO-1 Ameliorates the Deleterious Effects of Malaria During Early Pregnancy

**Figure 5:**
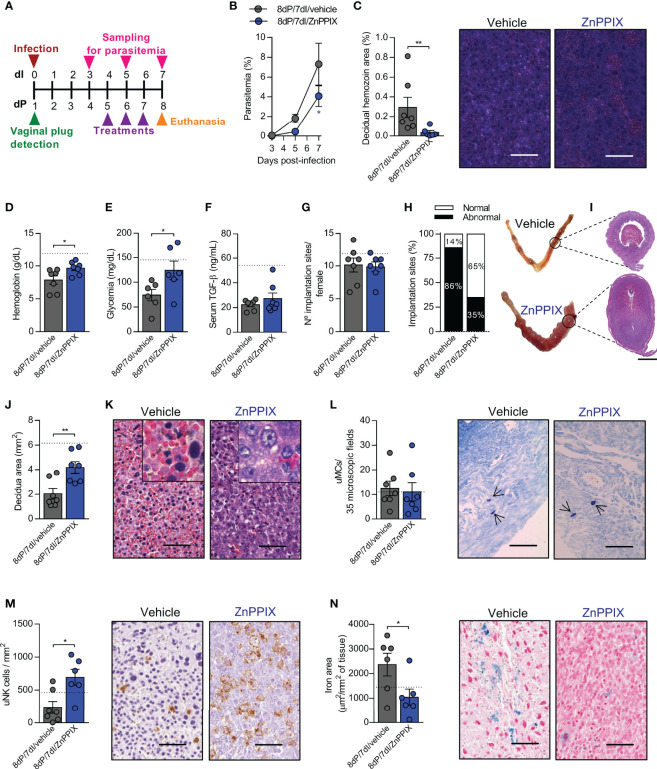
Inhibition of HO-1 ameliorates the deleterious effects of malaria during early pregnancy. **(A)** An experimental design for the pharmacological inhibition of HO-1 by ZnPPIX during malaria in early gestation was performed. **(B)** Parasitemia levels were estimated by flow cytometry. Each symbol represents the mean of all animals within the indicated group ± SEM. **(C)** Hemozoin was detected in decidual tissue using polarized light microscopy, scale bar 50 μm. **(D–F)** Hemoglobin, blood glucose and serum TGF-β levels were measured. **(G, H)** The number of implantations sites per female was counted **(G)** and classified as normal or abnormal according to macroscopic aspect **(H)** (n = 7 mice/group). **(I–K)** Uterine tissue was histologically processed and tissue sections were stained by H&E **(I)**, scale bar 1 mm. Decidual area was delimited **(J)** and implantation sites alterations were evaluated **(K)**, scale bar 50 μm. **(L)** uMCs were detected and quantified in Toluidine Blue stained implantation sites, scale bar 50 μm. **(M)** uNK cells were detected and quantified in DBA lectin-stained implantation sites, scale bar 50 μm. **(N)** Quantification of iron deposition and representative images of iron-stained tissue, scale bar 50 μm. Unless otherwise stated, each symbol represents the measure of one mouse and results represent mean ± SEM (n= 6-7 mice/group). The dashed lines indicate the mean results of pregnant uninfected and untreated mice for each parameter evaluated. Data was analyzed by Two-way ANOVA followed by Bonferroni’s *post-hoc* comparisons tests **(B)**, unpaired t-test **(D, E, G, J, L–N)** or Mann-Whitney test **(C, F, H)**. NP, non-pregnant; NI, non-infected; dP, days of pregnancy; dI, days of infection. *: p <0.05; **: p <0.01.

Since HO-1 is upregulated at the implantation sites during malaria in early pregnancy, we hypothesized that the adverse effects observed in these animals were induced by the increased activity of the enzyme. To investigate this, mice were infected on the day of vaginal plug detection, and treated with an inhibitor of HO-1 (ZnPPIX) or vehicle on gestational days 5, 6 and 7, as described in **Figure 5A**.

There was a reduction in parasitemia levels after 8dP and 7dI in mice treated with ZnPPIX in relation to those treated with vehicle (p=0.0399) (**Figure 5B**). Accordingly, reduced levels of hemozoin were detected in decidual tissue of mice treated with the HO-1 inhibitor (p=0.0047) (**Figure 5C**), demonstrating that ZnPPIX treatment is able to control the proliferation of the parasite and its accumulation in feto-maternal tissues in this gestational period. Furthermore, anemia (p=0.0317) and hypoglycemia (p=0.0453), but not the lower TGF-β levels, were prevented in infected pregnant mice treated with ZnPPIX in comparison with vehicle-treated mice (**Figures 5D–F**).

The treatments did not affect the number of embryos implanted in the uterus (**Figure 5G**); however, when the implantation sites were examined, it was noted that ZnPPIX treatment tended to improve the proportion of normal implantation sites in relation to those in infected mothers treated with vehicle (**Figure 5H**). Similarly, deciduas from infected mice treated with ZnPPIX were larger than those from mice treated with vehicle (p=0.0053) (**Figures 5I, J**). Most of the implantation sites of infected mice treated with vehicle displayed necrotic deciduas with extensive areas of hemorrhage, containing numerous infected red blood cells and decidual cells with pyknotic nuclei. In contrast, these histological changes were less common at implantation sites in infected mice treated with ZnPPIX (**Figure 5K**). On the other hand, although no differences were observed in the number of uMCs between the studied groups (**Figure 5L**), ZnPPIX treatment was able to preserve the number of uNK cells in the decidua of infected mice (p=0.0101) (**Figure 5M**).

Given that our results showed that the expression of HO-1 increases at implantation sites in animals in early pregnancy in parallel with iron overload in these tissues, we investigated whether inhibition of the enzyme would lead to less iron accumulation. As expected, we found that ZnPPIX treatment prevented iron overload at implantation sites (p=0.0426) (**Figure 5N**).

Additional experiments were also conducted to modulate the expression of HO-1 in mid and late pregnancy. Interestingly, ZnPPIX treatment was able to prevent infection-induced fetal growth restriction in late pregnancy (**Supplementary Figure S2**, **Table S1**). Despite of this, the overall results indicated that the protective effect of HO-1 modulation decreased as pregnancy progressed and that the main adverse effects of infection, associated with the activity of the enzyme, were restricted to early pregnancy (**Supplementary Figures S1–S3**).

### Malaria During Early Pregnancy Induces Oxidative Stress and Pro-Inflammatory Cytokine Production at the Implantation Sites, Both of Which Are Prevented by HO-1 Inhibition

**Figure 6:**
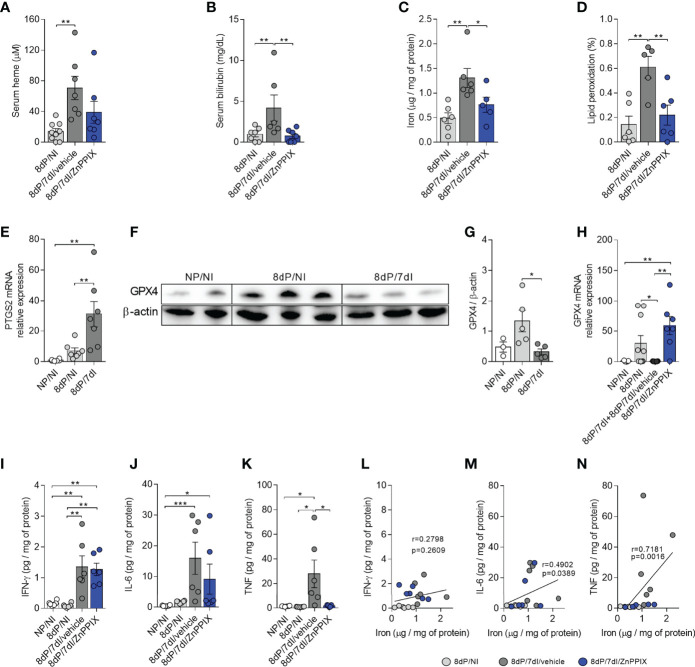
Infection-induced inflammation and oxidative stress are prevented by HO-1 inhibitory treatment. **(A, B)** Serum levels of heme (8dP/NI, n=9; 8dP/7dI/vehicle, n=7; 8dP/7dI/ZnPPIX, n=7) and bilirubin (8dP/NI, n=7; 8dP/7dI/vehicle, n=6; 8dP/7dI/ZnPPIX, n=7) were measured. **(C, D)** Iron (8dP/NI, n=6; 8dP/7dI/vehicle, n=6; 8dP/7dI/ZnPPIX, n=5) and lipid peroxidation levels (8dP/NI, n=6; 8dP/7dI/vehicle, n=5; 8dP/7dI/ZnPPIX, n=6) were quantified in tissue samples from implantation sites. **(E)** mRNA expression of PTGS2 in uterus/placenta was measured by qPCR normalizing to GAPDH expression and using NP/NI group as calibrator (NG/NI, n=6; 8dP/NI, n=7; 8dP/7dI, n=7). **(F, G)** Protein extracts from uterine samples were subjected to western blot analysis to determine the relative expression of GPX4 and β-actin proteins **(F)**. Quantitative analysis of the densitometric analysis of bands (NG/NI, n=3; 8dP/NI, n=5; 8dP/7dI, n=5) **(G)**. **(H)** mRNA expression of GPX4 in uterus/placenta was measured by qPCR normalizing to GAPDH expression and using NP/NI group as calibrator (NG/NI, n=5; 8dP/NI, n=10; 8dP/7dI/vehicle, n=7; 8dP/7dI/ZnPPIX, n=7). **(I–K)** Levels of the cytokines IFN-γ **(I)**, IL-6 **(J)** and TNF **(K)** were measured in uterine samples of indicated groups. **(L–N)** Pearson’s correlation **(L)** and Spearman’s correlation **(M, N)** analysis was performed between levels of IFN-γ, IL-6, TNF and iron concentrations in uterine tissue (n= 6 mice/group). Each symbol represents the measure of one mouse and results represent mean ± SEM. Data was analyzed by One-way ANOVA with Bonferroni’s *post-hoc* comparisons tests **(A, C, D, E, G, I, K)** or Kruskal-Wallis test with Dunn’s *post-hoc* comparisons tests **(B, H, J)**. NP, non-pregnant, NI, non-infected; dP, days of pregnancy; dI, days of infection. *: p <0.05; **: p <0.01; ***: p<0.001.

We found that infection led to an increase in serum heme concentrations (p=0.0018) and that the levels of this molecule appeared to be modulated in mice treated with ZnPPIX (**Figure 6A**). Similarly, while the infected animals presented high serum levels of bilirubin (p=0.0283) indicating an increase in HO-1 activity, mice treated with ZnPPIX presented lower bilirubin levels (p=0.0103), confirming inhibition of the enzyme’s activity (**Figure 6B**). Additionally, there was iron overload (p=0.0021) (**Figure 6C**) and induction of lipid peroxidation (p=0.0011) (**Figure 6D**) at the implantation sites during infection (p=0.0034), both of which were prevented by HO-1 inhibition treatment (p=0.0307 and 0.0042, respectively).

On the other hand, infection caused an increase in serum heme concentration in late pregnancy, but without causing iron accumulation or lipid peroxidation in this period; and in mid-pregnancy there were no changes in heme or bilirubin serum levels or tissue iron accumulation or lipid peroxidation. These data suggest that the pathogenetic mechanisms of the disease in these periods are not dependent on HO-1 activity, in contrast to early pregnancy (**Supplementary Figure S3**).

Since ferroptosis is a cell death process that involves the accumulation of lipid peroxides induced by iron (55, 56), we investigated whether this type of cell death occurs at the implantation sites of animals infected by *Plasmodium*, through the evaluation of the mRNA expression of PTGS2, which is increased during ferroptosis (51) and GPX4 protein and mRNA expression, which has anti-ferroptotic activity (17, 57). PTGS2 mRNA expression was increased in implantation sites in infected mice in comparison to uninfected pregnant (p=0.0043) and uninfected non-pregnant mice (p=0.0010) (**Figure 6E**). Furthermore, western blot analysis revealed a significant reduction in the protein levels of this enzyme at implantation sites in infected mice (p=0.0136) (**Figures 6F, G**). Therefore, these data indicate that cell death by ferroptosis could be taking place at implantation sites in infected mice. Accordingly, mRNA analysis showed that the transcript levels of GPX4 were lower at implantation sites in pregnant infected mice in relation to uninfected pregnant mice (p=0.0444) and that GPX4 mRNA is upregulated at implantation sites in infected mice treated with ZnPPIX when compared to uninfected non-pregnant mice (0.0051) and infected pregnant mice treated with vehicle (p=0.0013) (**Figure 6H**), suggesting that upregulation of GPX4 occurs at the transcriptional level, preventing ferroptosis.

T helper 1 (Th1) cytokines are produced during *Plasmodium* infection to induce mechanisms to control the proliferation of the parasite, but when they are produced in an exacerbated manner, they induce tissue damage (58). In order to evaluate local cytokine production in infected mice we measured the levels of IL-6, IFN-γ, and TNF in tissue homogenates of implantation sites. The results demonstrated that infection was able to induce an increase in the tissue levels of IFN-γ (p<0.0036) (**Figure 6I**) and TNF (p<0.0258) (**Figure 6K**) in infected mice treated with vehicle in relation to pregnant uninfected and non-pregnant uninfected mice as well as the levels of IL-6 in relation to non-pregnant uninfected mice (p<0.0006) (**Figure 6J**). Despite not controlling the increase in IL-6 and IFN-γ, inhibition of HO-1 significantly reduced TNF levels when compared with pregnant infected mice treated with vehicle (p=0.0392) (**Figure 6K**), suggesting that ZnPPIX is able to control, at least in part, infection-induced inflammation at implantation sites. In fact, we observed a positive correlation between the levels of IL-6 and TNF (**Figures 6M, N**), but not IFN-γ (**Figure 6L**), and the levels of iron at the implantation sites. In mid and late gestation, inflammation was driven by an increase in IFN-γ, but not TNF or IL-6 levels (**Supplementary Figure S3**).

## Discussion

This study found that *P. berghei* ANKA infection affected embryo viability in early and mid-pregnancy, while in late pregnancy infection compromised the success of the pregnancy by reducing fetal weight, as previously observed in other studies using various models of murine malaria in pregnancy, and reviewed by Barateiro et al. (34). In addition, it was demonstrated that poor pregnancy outcome and inflammation caused by malaria during early pregnancy are associated with increased expression and activity of HO-1 at the implantation sites, which lead to iron overload, lipid peroxidation and ferroptosis. HO-1 inhibition prevented most of the alterations provoked by the infection leading to a reduction in parasitemia levels as well as hemozoin accumulation, TNF levels, iron deposition and lipid peroxidation at implantation sites.

HO-1 has been shown to be elevated in plasma samples from infected pregnant women (59), and its mRNA expression was increased in the liver of pregnant BALB/c mice infected with *P. berghei* ANKA and conceptuses from C57BL/6 mice infected with *P. chabaudi* AS (60, 61). Furthermore, a previous study found that conceptuses of Swiss mice infected with *P. chabaudi* AS displayed an increase in HO-1 mRNA that was correlated with hemozoin density in placental tissues, showing that higher tissue hemozoin levels are related to higher HO-1 expression (62), which is consistent with our results.

The antimalarial effect exerted by ZnPPIX observed in this study could be explained in two ways: the first, could be due to competitive inhibition of HO-1, preventing the formation of heme catabolism products, including iron, which are necessary for the growth of the parasite (63, 64); the second could be attributed to its ability to prevent the process of hemozoin formation, similarly to the mechanism of action of many quinolones, such as chloroquine (65). It is possible that these mechanisms are restricted to the initial pregnancy stages due to the higher rate of parasitemia observed in the latter period in relation to the previous ones, exhausting the possible mechanisms of antiparasitic action exerted by ZnPPIX.

The reduction in uNK cells numbers during early pregnancy was associated with greater loss of embryonic viability in this study. The role of uNK cells in gestational malaria has not been well elucidated, since there is a lack of studies on these cells in murine models of malaria in pregnancy, and previous studies of placentas from women with malaria have focused on detecting NK cells in peripheral blood and blood from placental intervillous spaces (66, 67) or even placental tissue samples, although without quantifying NK cells in the decidua (68), which is the main tissue in which these cells are found (69). At delivery, the NK cell numbers were similar in placental blood from mothers with placental *Plasmodium falciparum* infection (PM+) and ones without placental malaria (PM-); however, IFN-γ production by NK cells in placental blood was higher in PM- individuals than in PM+ mothers, suggesting a protective role of placental IFN-γ-producing NK cells (67). Another study demonstrated that more NK cells were seen in *P. falciparum*-infected placentas from the third trimester than uninfected placentas, suggesting that NK cells in placentas may participate in mechanisms involved in the poor birth outcome associated with parasite infection (62). These studies were conducted using samples collected at parturition, at which time the number of these cells had declined (70, 71). In the present study it was shown for the first time that *P. berghei* infection induced a decrease in uNK cells in early gestation that was accompanied by poor pregnancy outcome, suggesting a protective role of this cell phenotype.

In rodent models, reduced numbers of uNK cells have been associated with decidual abnormalities and poor spiral artery remodeling, compromising the nutrient supply to the developing fetus (43, 72). HO-1 is required to support placentation and fetal development, an effect that can be mimicked by CO administration (73). HO-1 deficiency has been associated with reduced uNK cell numbers at early and mid-gestation implantation sites, these cells being restored by exogenous CO treatment (25). Surprisingly, we demonstrated that, during malaria infection, inhibition of HO-1 increases the number of uNK cells. In addition, it was verified that the decidua, the major site of uNK cell infiltration, was more preserved in ZnPPIX-treated than in infected untreated mice. Thus, although our results point to a protective role for uNK cells during *Plasmodium* infection in early pregnancy, more studies are necessary to elucidate the role of these cells in the context of malaria in pregnancy.

Examining the possible mechanisms involved in *Plasmodium* infection-induced pathology during pregnancy, Salifu et al. (59) found that infected pregnant women had increased serum levels of heme and HO-1, both of which increased further with iron supplementation. In addition, it was previously reported by Penha-Gonçalves et al. (33) that iron accumulation in trophoblast cells during experimental malaria was associated with fetal death. Accordingly, in the present investigation, an increase in serum heme, upregulation of HO-1 and iron overload at the feto–maternal interface were observed, but not in late gestation as observed by Penha-Gonçalves et al. (33). The different results observed in our experimental investigation could be related to the parasite load utilized and period of gestation and infection, since Penha-Gonçalves et al. (33) examined animals inoculated with a higher parasite load than that used in the present investigation and only in late gestation. Together, these results highlight the importance of studying iron status at the feto–maternal interface to prevent infection-induced adverse pregnancy outcomes.

Our data suggest that iron accumulation, consequent lipid peroxidation and the adverse outcomes caused by infection during early pregnancy are mediated by HO-1, since its inhibition led to an improvement in the gestational outcome associated with reduced iron levels, lipid peroxidation and TNF levels at the implantation sites in parallel with upregulation of GPX4 mRNA expression levels. One hypothesis is that once iron-induced oxidative stress is established, positive feedback occurs as HO-1 increases, which could also increase the accumulation of iron at the implantation sites. In this sense, Fisher et al. (74) demonstrated that iron overload causes increased expression of HO-1 in placental endothelial cells, and exposure to iron overload and inflammation have a synergistic effect on the induction of oxidative stress and cell death in a TNF-α-dependent manner. In fact, increased levels of circulating TNF have been associated with fetal loss in the first trimester of human pregnancy (75). TNF signaling induces the production of reactive oxygen species (ROS) capable of overwhelming endogenous cellular antioxidant mechanisms and decreasing GPX4 expression, leading to lipid peroxidation (76). Conversely, treatment with a GPX4 activator inhibited the TNF-induced activation of the NF-κB pathway promoting the resolution of inflammation and prevention of ferroptosis (77). Thus, in the present investigation embryonic loss could be due to a combination of iron accumulation and TNF production at implantation sites in infected mice during early gestation.

In the brain, aging was able to upregulate HO-1 expression, which in association with an inflammatory stimulus induced iron-mediated cell death and ferroptosis, while ZnPPIX treatment was able to decrease inflammatory markers (15). Moreover, HO-1 has been described as important for lipid peroxidation during ferroptosis. Treatment of fibrosarcoma cells with erastin, an ferroptosis inducer, increase mRNA and protein levels of HO-1 while treatment with ZnPPIX prevented erastin-induced cell death (78). Our results indicate that the adverse effects of *P. berghei* ANKA infection during early pregnancy are related to iron overload, lipid peroxidation and necrosis of the decidua provoked by the increased expression and activity of HO-1 at the implantation sites, suggesting ferroptotic cell death. Accordingly, higher levels of lipid peroxidation have been observed in placentas of BALB/c mice infected with *P. berghei* ANKA (79) and in serum samples of pregnant women with malaria when compared with non-infected pregnant women and infected non-pregnant women (80).

The reduction of lipid peroxides is essential for cell survival and, therefore, its iron-induced increase triggers cell death by ferroptosis. GPX4 activity protects cells against ferroptotic cell death and is important for normal mouse embryo development, since deficiency of this enzyme caused embryo death between 7.5– and 8.5-days post coitum (81, 82). We observed that at 8 days of pregnancy and 7 days of infection with *P. berghei* ANKA mice exhibited substantial embryo loss associated with high expression of HO-1 and hallmarks of ferroptotic cell death such as iron-induced lipid peroxidation, upregulation of *Ptgs2* and downregulation of *Gpx4*. On the other hand, HO-1 inhibition induced the opposite effects, improving pregnancy outcome and highlighting the importance of HO-1 regulation for prevention of ferroptosis at implantation sites and maintenance of pregnancy during malaria in early pregnancy.

In view of the well-known protective effects induced by HO-1 in different contexts and processes, as recently reviewed by Campbell et al. (83), including improvement in cerebral malaria (31) and infections by other pathogens during pregnancy (29, 30, 84), the finding that its increase could be related to tissue damage and, on the contrary, that its inhibition would be protective, was unexpected. However, it has been verified that the enzyme has cytoprotective activity when it increases less than five times the initial expression, while enhancement of HO-1 activity of more than 15 times is cytotoxic, inducing high levels of iron and lipid peroxidation. In this scenario, treatment with the HO-1 inhibitor tin mesoporphyrin (SnMP) decreased HO-1 activity to moderate levels (four to five times compared to the control), which decreased the levels of reactive iron and oxidative damage, highlighting that there is a beneficial HO-1 expression threshold related to the accumulation of reactive iron released in heme degradation and that this accumulation makes it unlikely that the excessive expression of HO-1 offers a protective response (85).

Our results showed that HO-1 expression and activity are enhanced in *Plasmodium* infection during early pregnancy and that ZnPPIX treatment seems to decrease the ability of HO-1 to exert its deleterious effect when in excessive amounts. On the other hand, HO-1 expression was unchanged and moderately increased in mid- and late-pregnancy periods, respectively, in agreement with the reduced protective effects of ZnPPIX as pregnancy progresses, which could mean that other mechanisms overlap in the pathogenesis of the disease in these periods, in addition to the effects of HO-1. The lack of conclusive changes in the expression of HO-1 in the mid and late pregnancy may be due to the strong predominance of the Th2 response profile in those periods, especially during mid-gestation (86). In contrast, early pregnancy is characterized by a Th1 response that favors embryo implantation, trophoblastic invasion, and vascular remodeling (86). Proinflammatory cytokines such as IL-1α and TNF are able to induce HO-1 expression (87). Interestingly, in the present work local levels of TNF were increased by infection in early pregnancy, time in which HO-1 overexpression and related pathological markers are observed, and decreased after HO-1 inhibitor treatment.

**Figure 7:**
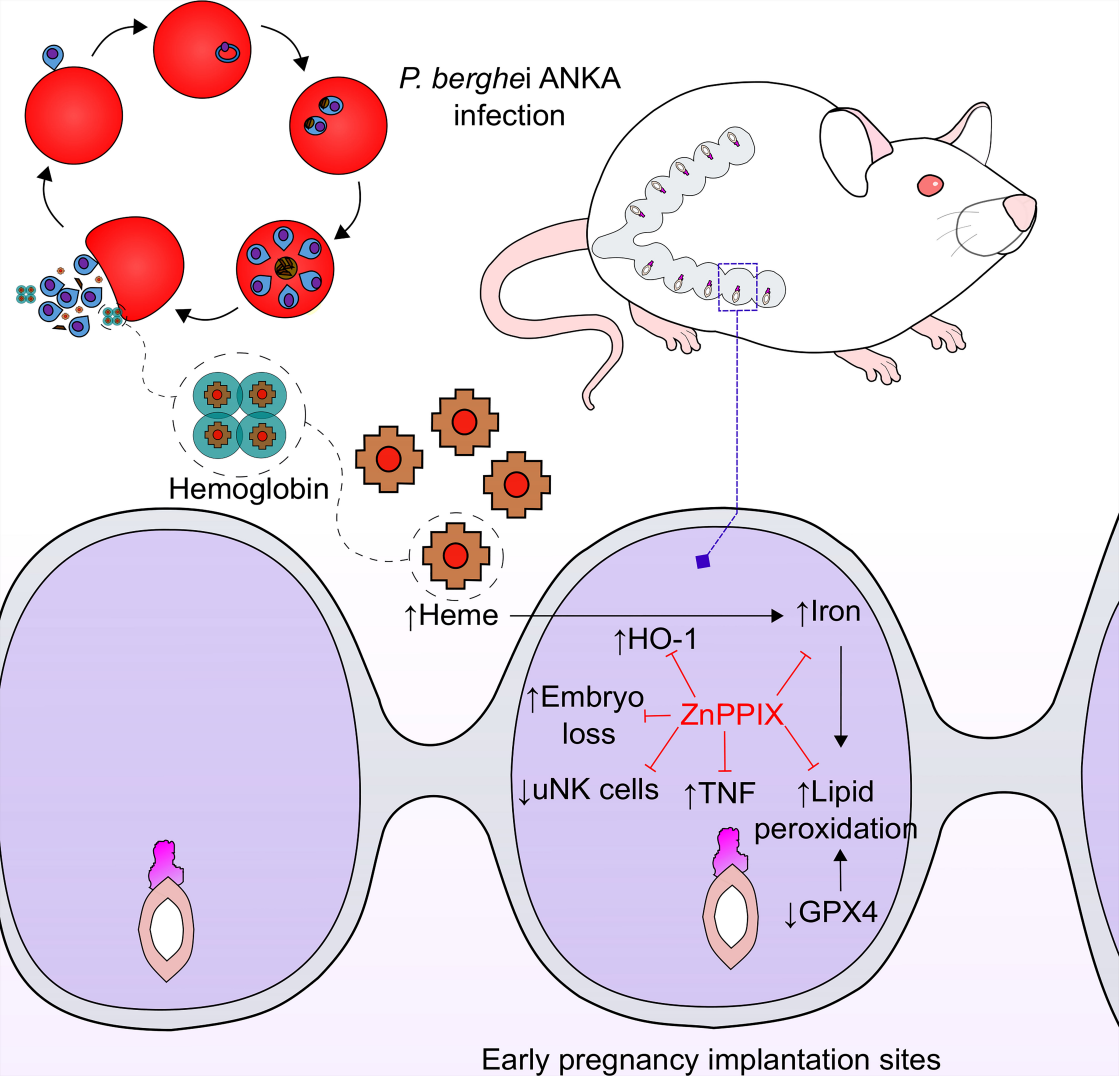
ZnPPIX beneficial effects in malaria during early pregnancy. HO-1 inhibition may have several beneficial effects in malaria during early pregnancy. Infection causes an increase in heme levels and induction of HO-1 in implantation sites leading to iron overload, lipid peroxidation and inflammation that ends up in embryo loss during early pregnancy, being these alterations prevented in ZnPPIX-treated mice. Moreover, ZnPPIX treatment preserves the number of uNK cells, that decrease in infected pregnant mice.

In summary, the data presented here suggest that infection with *P. berghei* ANKA causes hemolysis, releasing large amounts of heme that lead to upregulation of HO-1 at implantation sites in early pregnancy. This in turn leads to an increase in iron deposition in this tissue, causing oxidative stress *via* lipid peroxidation, and a decrease in *Gpx4* expression inducing ferroptosis, which together with the pro-inflammatory response mediated mainly by TNF lead to embryonic loss. Treatment with the enzyme inhibitor, ZnPPIX, modulates the enzyme’s activity and it can exert its beneficial effects, thus improving pregnancy outcome during infection (**Figure 7**). The results of the present investigation highlight the importance of the study of malaria in early gestation and open new perspectives in the therapeutic management of HO-1 in this context, especially in those women who receive iron supplementation.

## Materials and Methods

### Parasite Strain

The *P. berghei* ANKA strain expressing green fluorescent protein (GFP) was kindly provided by Dr. Ricardo Gazzinelli (Fiocruz, Brazil) and used to infect mice in this work. Parasites were maintained as frozen stocks and defrosted to infect BALB/c mice through inoculation of infected red blood cells (iRBCs) by the intraperitoneal (i.p.) route. To infect experimental animals, tail blood was collected from a previously infected mouse containing mainly parasites in ring or trophozoite stages, adjusting the inoculum to 5×10^4^ iRBCs per animal, which were injected by the i.p. route.

### Mice and Ethical Approval

Male and female BALB/c mice at 8- to 12-weeks-old were housed in specific-pathogen-free conditions and received water and food ad libitum. Animal experiments were approved by the Animal Experimental Ethics Committee (CEUA) of the Federal University of Uberlândia under protocol number 066/17 and performed according to institutional guidelines for animal ethics at the animal facilities of the Federal University of Uberlândia (Rede de Biotérios de Roedores - REBIR-UFU).

### HO-1 Inhibitor Treatment

Zinc protoporphyrin IX (ZnPPIX) (Cayman Chemical, Ann Arbor, MI, USA) a known inhibitor of HO-1 activity, was dissolved in NaOH 0.1M, diluted in PBS, and the pH was adjusted to 7.0 using HCl 1M, to be then administered in doses of 10 mg/kg/day by subcutaneous injection.

### Pregnancy Monitoring and Experimental Infection

BALB/c females were mated with males of the same lineage at a ratio of 2:1. Females were monitored daily and the day of vaginal plug detection was considered as the first day of pregnancy (dP). Pregnant mice were infected or not on gestational day (GD) 1, 5 and 12 and euthanized on GD 8, 12 and 19, respectively. Non-pregnant non-infected (NP/NI) and infected non-pregnant (NP/7dI) mice were used as controls when considered appropriate. All infected mice were euthanized after 7 days of infection (dI) (**Figures 1A**, **2A** and **3A**). For this purpose, mice were injected with the anesthetics Ketamine/Xylazine (Vetnil, Louveira, SP, Brazil) by the i.p. route, blood samples were collected from the retro-orbital plexus to obtain serum and from the tail for parasitemia and hemoglobin analysis, then euthanasia was performed by cervical dislocation.

The number of total, normal and abnormal implantation sites was documented. Implantation sites were considered abnormal when they presented a pale, hemorrhagic or necrotic appearance or a small size for gestational age, these features suggest that these implantation sites are being resorbed or have a high probability of undergoing embryonic resorption (**Supplementary Figure S4**). Additionally, placental and fetal weight were recorded when mice were euthanized on GD 19. Biometric measurements such as crown-rump length (CRL), snout-occipital distance (SOD), abdominal anteroposterior diameter (APD) and biparietal diameter (BPD) were also performed in fixed pups, as previously described (88, 89).

### Parasitemia Analysis

Tail blood samples were collected at 3, 5 and 7 days of infection, fixed in 4% formaldehyde in PBS and stored at 4 °C until processing for detection of infected red blood cells, through GFP expression by flow cytometry in FACSCanto II flow cytometer (BD Biosciences, San Jose, CA, USA). A total of 50,000 gated events were acquired and recorded for analysis of each sample using FlowJo v10 software (BD Biosciences).

### Biochemical Measurements

Serum glucose, serum bilirubin, blood hemoglobin and iron tissue levels were measured using commercial kits (Labtest Diagnóstica S.A. Belo Horizonte, MG, Brazil) according to the instructions of the kit for each parameter.

### Histological Processing

Whole implantation sites (8 and 12dP) and placentas (19dP) were fixed in 10% buffered formalin and embedded in paraffin. Tissue sections of 4 μm thick were made and stained with Hematoxylin and Eosin (H&E) for histological analysis. Additionally, Toluidine Blue staining was performed for uMC detection. These cells were quantified by examining 35 microscopic fields of the myometrium and endometrium layers of the uterus.

Images from tissues were obtained using an Aperio AT Turbo scanner (Leica Biosystems Inc., Buffalo Grove, IL, USA) that allowed the management of images of whole tissues for morphometric measurements of utero-placental layer areas or a Leica DM500 microscope connected to a Leica ICC50 digital camera (both Leica Microsystems, Wetzlar, Germany). These uterine and placental layers were delimited using QuPath software (90) (**Supplementary Figure S4**).

### Hemozoin Area Measurement

Hemozoin was detected in H&E-stained sections of uterus/placenta *via* polarized light microscopy. For this, three microscopic fields of decidual tissue per implantation site were randomly acquired in a Nikon Eclipse Ti-5 microscope and the area of hemozoin was estimated using Image J Fiji software (91). The results are expressed as the percentage of hemozoin area in relation to the total area of the image.

### Vascular Space Area Assessment

H&E-stained sections of placentas were used for estimation of vascular space areas as previously described (50). For this, five microscopic fields were randomly acquired from lateral and middle regions of the labyrinth layer of the placentas. These images were analyzed with Image J (NIH, Bethesda, MA, USA), in which an automated light analysis procedure was applied to ensure color and image quality standardization across sections. Then, a threshold color was used to cover the area corresponding to the vascular space lumen. The coverage of vascular space area was determined as the ratio between the number of pixels covered by the area defined by the threshold and, the total number of pixels in the image. Results were expressed as the mean of vascular space area of the placentas from each female.

### uNK Cells Detection

uNK cells were stained according to a previously described method (92). Tissue sections of implantation sites of mice in early and mid-pregnancy were deparaffinized, re-hydrated and incubated with 3% hydrogen peroxide for 30 min. Sections were washed in PBS and then incubated with 1% bovine serum albumin (BSA) (Sigma-Aldrich, St. Louis, MO, USA) in PBS for 30 min, followed by overnight incubation at 4°C with biotinylated-*Dolichos biflorus* (DBA) lectin (Sigma-Aldrich) diluted 1:150 in PBS. After that, sections were washed with PBS and incubated with avidin-biotin-peroxidase complex (ABC) (ABC kit, PK-4000; Vector Laboratories, Inc., Burlingame, CA, USA) at 37°C for 30 minutes and revealed with 3,3’-diaminobenzidine (Sigma-Aldrich) diluted in PBS containing hydrogen peroxide. The sections were counter-stained with Harris’ hematoxylin and mounted with Dammar resin. Regions of interest from mesometrial deciduas were delimited and uNK cells counted using Aperio ImageScope software (Leica Biosystems Inc.). Results were expressed as uNK cells/mm^2^.

### HO-1 and α-SMA Immunohistochemistry

Deparaffinized tissue sections were re-hydrated and submitted to antigenic retrieval in citrate buffer (pH 6.0) in a microwave oven for 7 min. Then slides were incubated with 3% hydrogen peroxide for 30 min. Sections were washed in PBS and nonspecific ligation sites were blocked using bovine serum albumin (BSA) 1% in PBS (α-SMA staining) or skimmed milk (Molico, Nestlé^®^, São Paulo, SP, Brazil) 2% in PBS (HO-1 staining) for 30 min, followed by overnight incubation at 4°C with mouse anti-α-actin antibody (1:500, Santa Cruz Biotechnology, Inc., Dallas, TX, USA) or rabbit anti-HO-1 antibody (1:300, Sigma-Aldrich) diluted in PBS. Subsequently, tissue sections were washed with PBS and incubated with goat anti-mouse (1:500, Sigma-Aldrich) or goat anti-rabbit (1:400, Sigma-Aldrich) antibodies at 37°C for 1 h and, then incubated with avidin-biotin-peroxidase complex (1:100; ABC kit, PK-4000, Vector Laboratories Inc.) at 37°C for 30 minutes and revealed with 3,3’-diaminobenzidine (Sigma-Aldrich) diluted in PBS containing hydrogen peroxide. The sections were counter-stained with Harris’ hematoxylin and mounted with Dammar resin.

For α-SMA staining quantification, images from the decidua were extracted using QuPath software (90) and analyzed with Image J Fiji software using the Colour Deconvolution method. After applying the “H DAB” vector option, α-SMA area was determined in the “Color 2 window” and normalized by the area of decidua analyzed.

HO-1^+^ cell counting was done in QuPath software by selecting the analyze, cell analysis, and positive cell detection buttons. Then, to segment the cells in the image the following parameters were set: Detection image: hematoxylin OD; requested pixel size:0.5 μm; background radius: 8 μm; median filter radius: 0 μm; sigma: 1.5 μm; minimum cell area: 10 μm^2^; maximum cell area: 400 μm^2^; threshold: 0.1; maximum background intensity: 2; Split by shape: on; Exclude DAB: off; Cell expansion: 5 μm; Include cell nucleus: on; Smooth boundaries: on; Make measurements: on; Score compartment: Cell DAB OD mean; Threshold 1+: 0.2; single threshold: on. The number of HO-1^+^ cells was normalized according to the total area of tissue analyzed (**Supplementary Figure S4**).

### Protein Extraction and Expression Analysis by Western Blot

Tissue samples of implantation sites of mice at 8dP were weighed and homogenized in RIPA buffer (Tris 0.05M; NaCl 0.150M; Triton X-100 0.2%; sodium deoxycholate 0.2%; SDS 0.1%) with 10 mM EDTA, containing protease inhibitor cocktail (cOmplete ™, Roche Diagnostics, Mannheim, Germany), 1 mM Na_3_VO_4_ and 25 mM NaF. The samples were homogenized in a proportion of 1 mL of extraction buffer for each 200 mg of tissue. After incubation in ice for 30 minutes, the samples were centrifuged at 15000 ×g for 30 minutes at 4°C, the supernatants were collected, protein concentrations were measured using the Bradford method (93) and these samples were stored at -80 °C until the moment of use. Protein extracts (60 µg protein/lane) were submitted to electrophoresis in polyacrylamide gels (SDS-PAGE) at 12% and transferred to polyvinylidene fluoride (PVDF) membranes (Immobilon^®^-FL, Millipore Corporation, Billerica, USA). The membranes were subsequently blocked with skimmed milk (Itambé, Belo Horizonte, MG, Brazil) 5% in blotting buffer (Tris buffer/Tween 20 0.1%) for 1 h, incubated overnight with mouse anti-GPX4 antibody (1:1000, Santa Cruz Biotechnology, Inc.) or mouse anti-β-actin antibody (1:1000; Sigma-Aldrich), both diluted in blotting buffer containing 3% BSA and 0.02% sodium azide. After that, membranes were washed three times of 10 minutes each in blotting buffer and incubated with peroxidase-conjugated goat anti-mouse secondary antibody diluted in 2% skimmed milk (1: 3000; Sigma-Aldrich) for 2 h. Finally, membranes were washed and the reactions were developed using a chemiluminescent substrate (ECL) (SuperSignal^®^ West Pico kit, Thermo Fisher Scientific, USA). Membranes photodocumentation was performed on translluminator ChemiDoc™ MP Imaging System (Bio-Rad Laboratories, Hercules, CA, USA). Densitometric analysis was performed using Image Lab Software (Bio-Rad Laboratories). Data were expressed as relative expression of the interest protein (GPX4) in relation to endogenous protein control (β-actin).

### Cytokine Analysis

Tissue samples were homogenized in 5 mM EDTA/PBS buffer containing protease inhibitor cocktail (cOmplete ™, Roche) using a Turrax T10 (IKA Works GmbH & Co. KG-Staufen– Germany) homogenizer (200 mg of tissue in 1 mL of buffer). Later, samples were centrifuged at 3000 × g for 10 min at 4°C. After that, supernatants were collected, aliquoted and stored at -80°C until use. IL-6, TNF and IFN-γ concentrations were measured utilizing a mouse Th1/Th2/Th17 CBA kit (BD Biosciences) following manufacturer’s instructions. Data were acquired on a FACSCanto II Flow Cytometer (BD Biosciences) and analyzed with FACSDiva software (BD Biosciences). Pierce™ BCA protein assay kit (Thermo Fisher Scientific) was employed to determine the total protein concentrations that were used for results normalization. The cytokine theoretical limits of detection (pg/mL) are: IL-6, 1.4; TNF, 0.9; IFN-γ, 0.5.

TGF-β levels were measured in serum samples using a sandwich ELISA kit following the manufacturer’s instructions (R&D Systems, Minneapolis, MN, USA). Detection limit for this assay was 15,6 pg/mL.

### Heme Quantification

Heme measurements in tissue samples were performed as described by (94) using 1-Step™ Turbo TMB (Thermo Fisher Scientific), a mixture of TMB and a stabilized hydrogen peroxide, due to the pseudoperoxidase characteristics of heme. Standard hemin chloride (Sigma-Aldrich) concentrations were used to extrapolate the heme concentrations in experimental samples.

### Iron Deposition Assessment

Iron was measured in supernatant of tissue homogenates using a commercial kit (Labtest Diagnóstica S.A.) following the manufacturer’s instructions and normalized by the total protein concentration. Additionally, iron deposition was evaluated in whole tissue sections stained with Perls’ Prussian Blue. Images of whole tissue were extracted utilizing the QuPath software and blue staining area was quantified making use of the Color Threshold of Image J Fiji. Results were normalized according to the total section area.

### Lipid Peroxidation Test

Lipid peroxidation was measured according to the thiobarbituric acid reactive substances (TBARS) method. Samples of tissue homogenates were incubated with a mixture of 10% trichloroacetic acid and 0.67% thiobarbituric acid for 2h at 40°C, after that n-butanol was added and mixed. Subsequently, the samples were centrifuged at 3000 ×g for 15 min, upper phase was collected and the fluorescence was measured at 515 nm (excitation) and 553 (emission) (Molecular Devices, Menlo Park, CA, USA). The percentage of lipid peroxidation was calculated as previously described (95).

### RNA Extraction and Quantitative PCR

Frozen pieces of uterus/placenta were mechanically pulverized in liquid nitrogen. Total RNA was extracted using TRIzol^®^ (Ambion^®^, Life Technologies, Carlsbad, CA, USA) and quantified by measuring the absorbance at 260 nm on GeneQuant spectrophotometer (GE Healthcare). Samples were treated with DNAse (Invitrogen, Thermo Fisher Scientific) and transcribed into complementary DNA (cDNA). Reactions were performed using 1 µg of RNA and reverse transcriptase ImProm-II™ (Promega, Madison, WI, USA) on Arktik thermocycler (Thermo Scientific). The cDNA was amplified in an ABI PRISM-7500 sequence detection system (Applied Biosystems, USA) using GoTaq^®^ qPCR Master Mix (Promega) and gene-specific primers for HO-1, PTGS2, GPX4 and GAPDH. Cycle threshold data were normalized to the expression of GAPDH and analyzed using 2^−ΔΔCt^ method (96). The sequences for each forward (Fw) and reverse (Rv) primers used in this study were as follows: HO-1 Fw 5′-GCTGGTGATGGCTTCCTTGT-3′, Rv 5′-GTGGGGCATAGACTGGGTTCT-3′; GPX4 Fw 5’- GCAACCAGTTTGGGAGGCAGGAG-3’, Rv 5’- GAAGCGCTATGGTCCCATGGAGG-3’; PTGS2 Fw 5′- CTGCGCCTTTTCAAGGATGG-3′, Rv 5′- GGGGATACACCTCTCCACCA-3′; GAPDH Fw 5′-GGAGAAACCTGCCAAGTA TGATG-3′, Rv 5′-CAGTGTAGCCCAAGATGCCC-3′.

### Statistical Analysis

Statistical analysis and graphs were performed using GraphPad Prism version 7.0 (GraphPad Software version 7.0, San Diego, CA, USA). Statistical differences between groups were determined by One-way ANOVA and Two-way ANOVA followed by Bonferroni’s *post hoc* test, as well as unpaired t-test for normally distributed values. Non-normally distributed values were analyzed by Mann-Whitney test or Kruskal Wallis test followed by Dunn’s *post hoc* test. Values of p < 0.05 were considered statistically significant.

## Data Availability Statement

The original contributions presented in the study are included in the article/**Supplementary Material**. Further inquiries can be directed to the corresponding author.

## Ethics Statement

The animal study was reviewed and approved by Animal Experimental Ethics Committee (CEUA) of the Federal University of Uberlândia.

## Author Contributions

NS conceived the idea. YC performed all the experimental procedures, statistical analysis, figure design and wrote the manuscript. MA and YC performed western blot analysis. MP, EA, AD-R and MA provided technical help in the processing of samples for mRNA expression measurement. EA and MA helped in the cytokine and biochemical measurements. AD-R provided assistance with animal handling and histological analysis. RF and FE contributed with measurement of lipid peroxidation. NS provided financial support, supervised the experiments, analysed the data and critically reviewed the manuscript. All the authors critically reviewed the manuscript.

## Funding

This work was supported by Fundação de Amparo à Pesquisa do Estado de Minas Gerais (FAPEMIG), and Conselho Nacional de Desenvolvimento Científico e Tecnológico (CNPq). In addition, this study was financed in part by the Coordenação de Aperfeiçoamento de Pessoal de Nível Superior, Brazil (CAPES), Finance Code 001. N.M. Silva is research fellow from CNPq.

## Conflict of Interest

The authors declare that the research was conducted in the absence of any commercial or financial relationships that could be construed as a potential conflict of interest.

## Publisher’s Note

All claims expressed in this article are solely those of the authors and do not necessarily represent those of their affiliated organizations, or those of the publisher, the editors and the reviewers. Any product that may be evaluated in this article, or claim that may be made by its manufacturer, is not guaranteed or endorsed by the publisher.

## References

[1] WHO. World Malaria Report, World Health Organization (2019). Available at: https://www.who.int/publications-detail-redirect/9789241565721 (Accessed February 11, 2022).

[2] Desai M ter Kuile FO Nosten F McGready R Asamoa K Brabin B. Epidemiology and Burden of Malaria in Pregnancy. Lancet Infect Dis (2007) 7:2. doi: 10.1016/s1473-3099(07)70021-x 17251080

[3] Dutra FF Bozza MT. Heme on Innate Immunity and Inflammation. Front Pharmacol (2014) 5:115. doi: 10.3389/fphar.2014.00115 24904418 PMC4035012

[4] Pádua TA Souza MC. Heme on Pulmonary Malaria: Friend or Foe? Front Immunol (2020) 11:1835. doi: 10.3389/fimmu.2020.01835 32983096 PMC7477073

[5] Slater AF. Malaria Pigment. Exp Parasitol (1992) 74:3. doi: 10.1016/0014-4894(92)90162-4 1582489

[6] Gozzelino R Jeney V Soares MP. Mechanisms of Cell Protection by Heme Oxygenase-1. Annu Rev Pharmacol Toxicol (2010) 50. doi: 10.1146/annurev.pharmtox.010909.105600 20055707

[7] Tenhunen R Marver HS Schmid R. The Enzymatic Conversion of Heme to Bilirubin by Microsomal Heme Oxygenase. Proc Natl Acad Sci USA (1968) 61:2. doi: 10.1073/pnas.61.2.748 PMC225223 4386763

[8] Duvigneau JC Esterbauer H Kozlov AV. Role of Heme Oxygenase as a Modulator of Heme-Mediated Pathways. Antioxidants (Basel) (2019) 8:10. doi: 10.3390/antiox8100475 PMC6827082 31614577

[9] Stocker R Glazer AN Ames BN. Antioxidant Activity of Albumin-Bound Bilirubin. Proc Natl Acad Sci USA (1987) 84:16. doi: 10.1073/pnas.84.16.5918 3475708 PMC298974

[10] Zenclussen AC Schumacher A Zenclussen ML Wafula P Volk HD. Immunology of Pregnancy: Cellular Mechanisms Allowing Fetal Survival Within the Maternal Uterus. Expert Rev Mol Med (2007) 9:10. doi: 10.1017/s1462399407000294 17462112

[11] Schumacher A Zenclussen AC. Effects of Heme Oxygenase-1 on Innate and Adaptive Immune Responses Promoting Pregnancy Success and Allograft Tolerance. Front Pharmacol (2015) 5:288. doi: 10.3389/fphar.2014.00288 25610397 PMC4285018

[12] Zenclussen ML Linzke N Schumacher A Fest S Meyer N Casalis PA. Heme Oxygenase-1 is Critically Involved in Placentation, Spiral Artery Remodeling, and Blood Pressure Regulation During Murine Pregnancy. Front Pharmacol (2015) 5:291. doi: 10.3389/fphar.2014.00291 25628565 PMC4292788

[13] Amaral EP Costa DL Namasivayam S Riteau N Kamenyeva O Mittereder L. A Major Role for Ferroptosis in Mycobacterium Tuberculosis-Induced Cell Death and Tissue Necrosis. J Exp Med (2019) 216:556–70. doi: 10.1084/jem.20181776 PMC6400546 30787033

[14] Costa DL Amaral EP Namasivayam S Mittereder LR Fisher L Bonfim CC. Heme Oxygenase-1 Inhibition Promotes Ifnγ- and NOS2-Mediated Control of Mycobacterium Tuberculosis Infection. Mucosal Immunol (2021) 14:253–66. doi: 10.1038/s41385-020-00342-x PMC7796944 32862202

[15] Fernández-Mendívil C Luengo E Trigo-Alonso P García-Magro N Negredo P López MG. Protective Role of Microglial HO-1 Blockade in Aging: Implication of Iron Metabolism. Redox Biol (2021) 38:101789. doi: 10.1016/j.redox.2020.101789 33212416 PMC7680814

[16] Duvigneau JC Piskernik C Haindl S Kloesch B Hartl RT Hüttemann M. A Novel Endotoxin-Induced Pathway: Upregulation of Heme Oxygenase 1, Accumulation of Free Iron, and Free Iron-Mediated Mitochondrial Dysfunction. Lab Invest (2008) 88:70–7. doi: 10.1038/labinvest.3700691 17982471

[17] Imai H Matsuoka M Kumagai T Sakamoto T Koumura T. “Lipid Peroxidation-Dependent Cell Death Regulated by GPx4 and Ferroptosis,”. In: Nagata S Nakano H, editors. Apoptotic and Non-Apoptotic Cell Death. Switzerland: Springer, Cham (2017). p. 143–70. doi: 10.1007/82_2016_508 28204974

[18] Hirschhorn T Stockwell BR. The Development of the Concept of Ferroptosis. Free Radic Biol Med (2019) 133. doi: 10.1016/j.freeradbiomed.2018.09.043 PMC6368883 30268886

[19] Brigelius-Flohé R Maiorino M. Glutathione Peroxidases. Biochim Biophys Acta (2013) 1830:5. doi: 10.1016/j.bbagen.2012.11.020 23201771

[20] Maurya VK DeMayo FJ Lydon JP. Illuminating the "Black Box" of Progesterone-Dependent Embryo Implantation Using Engineered Mice. Front Cell Dev Biol (2021) 9:640907. doi: 10.3389/fcell.2021.640907 33898429 PMC8058370

[21] Pang SC Janzen-Pang J Tse M Croy B Lima PDA. “The Cycling and Pregnant Mouse: Gross Anatomy,”. In: Croy A Yamada AT DeMayo FJ Adamson S, editors. The Guide to Investigation of Mouse Pregnancy. Cambridge, MA: Academic Press Elsevier (2014). p. 3–19. doi: 10.1016/B978-0-12-394445-0.00001-1

[22] Erlebacher A. “Leukocyte Population Dynamics and Functions at the Maternal–Fetal Interface,”. In: Croy A Yamada AT DeMayo FJ Adamson S, editors. The Guide to Investigation of Mouse Pregnancy. Cambridge, MA: Academic Press Elsevier (2014). p. 227–42. doi: 10.1016/B978-0-12-394445-0.00019-9

[23] Moffett-King A. Natural Killer Cells and Pregnancy. Nat Rev Immunol (2002) 2:9. doi: 10.1038/nri886 12209134

[24] Simmons DG. “Postimplantation Development of the Chorioallantoic Placenta,”. In: Croy A Yamada AT DeMayo FJ Adamson S, editors. The Guide to Investigation of Mouse Pregnancy. Cambridge, MA: Academic Press Elsevier (2014). p. 143–61. doi: 10.1016/B978-0-12-394445-0.00012-6

[25] Linzke N Schumacher A Woidacki K Croy BA Zenclussen AC. Carbon Monoxide Promotes Proliferation of Uterine Natural Killer Cells and Remodeling of Spiral Arteries in Pregnant Hypertensive Heme Oxygenase-1 Mutant Mice. Hypertension (2014) 63:3. doi: 10.1161/HYPERTENSIONAHA.113.02403 24366077

[26] Zenclussen AC Sollwedel A Bertoja AZ Gerlof K Zenclussen ML Woiciechowsky C. Heme Oxygenase as a Therapeutic Target in Immunological Pregnancy Complications. Int Immunopharmacol (2005) 5:1. doi: 10.1016/j.intimp.2004.09.011 15589458

[27] Zhao H Wong RJ Kalish FS Nayak NR Stevenson DK. Effect of Heme Oxygenase-1 Deficiency on Placental Development. Placenta (2009) 30:10. doi: 10.1016/j.placenta.2009.07.012 PMC2771543 19699520

[28] Zhao H Azuma J Kalish F Wong RJ Stevenson DK. Maternal Heme Oxygenase 1 Regulates Placental Vasculature Development *via* Angiogenic Factors in Mice. Biol Reprod (2011) 85:5. doi: 10.1095/biolreprod.111.093039 PMC3197918 21778140

[29] Tachibana M Watanabe K Yamasaki Y Suzuki H Watarai M. Expression of Heme Oxygenase-1 is Associated With Abortion Caused by *Brucella Abortus* Infection in Pregnant Mice. Microb Pathog (2008) 45:2. doi: 10.1016/j.micpath.2008.04.002 18501554

[30] Tachibana M Hashino M Nishida T Shimizu T Watarai M. Protective Role of Heme Oxygenase-1 in *Listeria Monocytogenes*-Induced Abortion. PloS One (2011) 6:9. doi: 10.1371/journal.pone.0025046 PMC3174987 21949846

[31] Pamplona A Ferreira A Balla J Jeney V Balla G Epiphanio S. Heme Oxygenase-1 and Carbon Monoxide Suppress the Pathogenesis of Experimental Cerebral Malaria. Nat Med (2007) 13:6. doi: 10.1038/nm1586 17496899

[32] Pereira ML Ortolan LS Sercundes MK Debone D Murillo O Lima FA. Association of Heme Oxygenase 1 With Lung Protection in Malaria-Associated ALI/ARDS. Mediators Inflamm (2016) 2016:4158698. doi: 10.1155/2016/4158698 27974865 PMC5126464

[33] Penha-Gonçalves C Gozzelino R de Moraes L. Iron Overload in *Plasmodium Berghei*-Infected Placenta as a Pathogenesis Mechanism of Fetal Death. Front Pharmacol (2014) 5:155. doi: 10.3389/fphar.2014.00155 25071574 PMC4077027

[34] Barateiro A Pereira MLM Epiphanio S Marinho CRF. Contribution of Murine Models to the Study of Malaria During Pregnancy. Front Microbiol (2019) 10:1369. doi: 10.3389/fmicb.2019.01369 31275284 PMC6594417

[35] Boonyapranai K Surinkaew S Somsak V Rattanatham R. Protective Effects of *Gymnema Inodorum* Leaf Extract on *Plasmodium Berghei*-Induced Hypoglycemia, Dyslipidemia, Liver Damage, and Acute Kidney Injury in Experimental Mice. J Parasitol Res (2021) 2021:1896997. doi: 10.1155/2021/1896997 34552764 PMC8452429

[36] Chaiyaroj SC Rutta AS Muenthaisong K Watkins P Na Ubol M Looareesuwan S. Reduced Levels of Transforming Growth Factor-Beta1, Interleukin-12 and Increased Migration Inhibitory Factor are Associated With Severe Malaria. Acta Trop (2004) 89:3. doi: 10.1016/j.actatropica.2003.10.010 14744558

[37] Abrams ET Kwiek JJ Mwapasa V Kamwendo DD Tadesse E Lema VM. Malaria During Pregnancy and Foetal Haematological Status in Blantyre, Malawi. Malar J (2005) 4:39. doi: 10.1186/1475-2875-4-39 16122391 PMC1232864

[38] Ali AA Elhassan EM Magzoub MM Elbashir MI Adam I. Hypoglycaemia and Severe *Plasmodium Falciparum* Malaria Among Pregnant Sudanese Women in an Area Characterized by Unstable Malaria Transmission. Parasit Vectors (2011) 4:88. doi: 10.1186/1756-3305-4-88 21605445 PMC3118382

[39] Botelho-Nevers E Laurencin S Delmont J Parola P. Imported Malaria in Pregnancy: A Retrospective Study of 18 Cases in Marseilles, France. Ann Trop Med Parasitol (2005) 99:7. doi: 10.1179/136485905X65099 16212804

[40] Jiménez BC Cuadros-Tito P Ruiz-Giardin JM Rojo-Marcos G Cuadros-González J Canalejo E. Imported Malaria in Pregnancy in Madrid. Malar J (2012) 11:112. doi: 10.1186/1475-2875-11-112 22494463 PMC3350381

[41] Finn CA McLaren A. A Study of the Early Stages of Implantation in Mice. J Reprod Fertil (1967) 13:2. doi: 10.1530/jrf.0.0130259 4164526

[42] Bolon B. “Pathology Analysis of the Placenta,”. In: Croy A Yamada AT DeMayo FJ Adamson S, editors. The Guide to Investigation of Mouse Pregnancy. Cambridge, MA: Academic Press Elsevier (2014). p. 175–88. doi: 10.1016/B978-0-12-394445-0.00014-X

[43] Meyer N Woidacki K Knöfler M Meinhardt G Nowak D Velicky P. Chymase-Producing Cells of the Innate Immune System are Required for Decidual Vascular Remodeling and Fetal Growth. Sci Rep (2017) 7:45106. doi: 10.1038/srep45106 28327604 PMC5361184

[44] Meyer N Woidacki K Maurer M Zenclussen AC. Safeguarding of Fetal Growth by Mast Cells and Natural Killer Cells: Deficiency of One Is Counterbalanced by the Other. Front Immunol (2017) 8:711. doi: 10.3389/fimmu.2017.00711 28670317 PMC5472686

[45] Brosens I Robertson WB Dixon HG. The Physiological Response of the Vessels of the Placental Bed to Normal Pregnancy. J Pathol Bacteriol (1967) 93:2. doi: 10.1002/path.1700930218 6054057

[46] Cartwright JE Fraser R Leslie K Wallace AE James JL. Remodelling at the Maternal-Fetal Interface: Relevance to Human Pregnancy Disorders. Reproduction (2010) 140:6. doi: 10.1530/REP-10-0294 20837731

[47] Woods L Perez-Garcia V Hemberger M. Regulation of Placental Development and Its Impact on Fetal Growth-New Insights From Mouse Models. Front Endocrinol (Lausanne) (2018) 9:570. doi: 10.3389/fendo.2018.00570 30319550 PMC6170611

[48] Kieckbusch J Balmas E Hawkes DA Colucci F. Disrupted PI3K P110δ Signaling Dysregulates Maternal Immune Cells and Increases Fetal Mortality In Mice. Cell Rep (2015) 13:12. doi: 10.1016/j.celrep.2015.11.050 PMC4700049 26711346

[49] Pawlak JB Bálint L Lim L Ma W Davis RB Benyó Z. Lymphatic Mimicry in Maternal Endothelial Cells Promotes Placental Spiral Artery Remodeling. J Clin Invest (2019) 129:11. doi: 10.1172/JCI120446 PMC6819089 31415243

[50] Neres R Marinho CRF Gonçalves LA Catarino MB Penha-Gonçalves C. Pregnancy Outcome and Placenta Pathology in *Plasmodium Berghei* ANKA Infected Mice Reproduce the Pathogenesis of Severe Malaria in Pregnant Women. PloS One (2008) 3:2. doi: 10.1371/journal.pone.0001608 PMC2229663 18270595

[51] De Beaudrap P Turyakira E White LJ Nabasumba C Tumwebaze B Muehlenbachs A. Impact of Malaria During Pregnancy on Pregnancy Outcomes in a Ugandan Prospective Cohort With Intensive Malaria Screening and Prompt Treatment. Malaria J (2013) 12:139. doi: 10.1186/1475-2875-12-139 PMC3642015 23617626

[52] Schmiegelow C Minja D Oesterholt M Pehrson C Suhrs HE Boström S. Malaria and Fetal Growth Alterations in the 3(Rd) Trimester of Pregnancy: A Longitudinal Ultrasound Study. PloS One (2013) 8:1. doi: 10.1371/journal.pone.0053794 PMC3543265 23326508

[53] Gallardo V González M Toledo F Sobrevia L. Role of Heme Oxygenase 1 and Human Chorionic Gonadotropin in Pregnancy Associated Diseases. Biochim Biophys Acta Mol Basis Dis (2020) 1866:2. doi: 10.1016/j.bbadis.2019.07.016 31376481

[54] Hunt NH Stocker R. Heme Moves to Center Stage in Cerebral Malaria. Nat Med (2007) 13:6. doi: 10.1038/nm0607-667 17554329

[55] Dixon SJ Lemberg KM Lamprecht MR Skouta R Zaitsev EM Gleason CE. Ferroptosis: An Iron-Dependent Form of Nonapoptotic Cell Death. Cell (2012) 149:5. doi: 10.1016/j.cell.2012.03.042 PMC3367386 22632970

[56] Yang WS Stockwell BR. Ferroptosis: Death by Lipid Peroxidation. Trends Cell Biol (2016) 26:3. doi: 10.1016/j.tcb.2015.10.014 26653790 PMC4764384

[57] Yang WS SriRamaratnam R Welsch ME Shimada K Skouta R Viswanathan VS. Regulation of Ferroptotic Cancer Cell Death by GPX4. Cell (2014) 156:1–2. doi: 10.1016/j.cell.2013.12.010 PMC4076414 24439385

[58] Deroost K Pham TT Opdenakker G Van den Steen PE. The Immunological Balance Between Host and Parasite in Malaria. FEMS Microbiol Rev (2016) 40:2. doi: 10.1093/femsre/fuv046 26657789

[59] Salifu H Wilson NO Liu M Dickinson-Copeland C Yatich N Keenan J. Iron Supplementation Alters Heme and Heme Oxygenase 1 (HO-1) Levels In Pregnant Women in Ghana. SOJ Microbiol Infect Dis (2016) 4:3. doi: 10.15226/sojmid/4/2/00154 PMC5258109 28124024

[60] Najjar N McColl ER Weckman A Kain KC Piquette-Miller M. Dysregulation of Solute Carrier Transporters in Malaria-Infected Pregnant Mice. Parasite Immunol (2019) 41:4. doi: 10.1111/pim.12614 30703256

[61] Sarr D Cooper CA Bracken TC Martinez-Uribe O Nagy T Moore JM. Oxidative Stress: A Potential Therapeutic Target in Placental Malaria. Immunohorizons (2017) 1:4. doi: 10.4049/immunohorizons.1700002 PMC5589203 28890952

[62] Morffy Smith CD Russ BN Andrew AK Cooper CA Moore JM. A Novel Murine Model for Assessing Fetal and Birth Outcomes Following Transgestational Maternal Malaria Infection. Sci Rep (2019) 9:1. doi: 10.1038/s41598-019-55588-8 31862902 PMC6925284

[63] Gabay T Ginsburg H. Hemoglobin Denaturation and Iron Release in Acidified Red Blood Cell Lysate–a Possible Source of Iron for Intraerythrocytic Malaria Parasites. Exp Parasitol (1993) 77:3. doi: 10.1006/expr.1993.1084 8224082

[64] Maines MD. Zinc . Protoporphyrin is a Selective Inhibitor of Heme Oxygenase Activity in the Neonatal Rat. Biochim Biophys Acta (1981) 673:3. doi: 10.1016/0304-4165(81)90465-7 6894392

[65] Iyer JK Shi L Shankar AH Sullivan DJ Jr. Zinc Protoporphyrin IX Binds Heme Crystals to Inhibit the Process of Crystallization in Plasmodium Falciparum. Mol Med (2003) 9:5–8. doi: 10.2119/2003-00010.sullivan PMC1430826 14571325

[66] Ordi J Menendez C Ismail MR Ventura PJ Palacín A Kahigwa E. Placental Malaria is Associated With Cell-Mediated Inflammatory Responses With Selective Absence of Natural Killer Cells. J Infect Dis (2001) 183:7. doi: 10.1086/319295 11237836

[67] Othoro C Moore JM Wannemuehler KA Moses S Lal A Otieno J. Elevated Gamma Interferon-Producing NK Cells, CD45RO Memory-Like T Cells, and CD4 T Cells are Associated With Protection Against Malaria Infection in Pregnancy. Infect Immun (2008) 76:4. doi: 10.1128/IAI.01420-07 PMC2292852 18250175

[68] Sartelet H Schleiermacher D Le-Hesran JY Graesslin O Gaillard D Fe M. Less HLA-G Expression in *Plasmodium Falciparum*-Infected Third Trimester Placentas is Associated With More Natural Killer Cells. Placenta (2005) 26:6. doi: 10.1016/j.placenta.2004.08.006 15950065

[69] Vento-Tormo R Efremova M Botting RA Turco MY Vento-Tormo M Meyer KB. Single-Cell Reconstruction of the Early Maternal-Fetal Interface in Humans. Nature (2018) 563:7731. doi: 10.1038/s41586-018-0698-6 PMC7612850 30429548

[70] Gomez-Lopez N Guilbert LJ Olson DM. Invasion of the Leukocytes Into the Fetal-Maternal Interface During Pregnancy. J Leukoc Biol (2010) 88:4. doi: 10.1189/jlb.1209796 20519637

[71] St-Germain LE Castellana B Baltayeva J Beristain AG. Maternal Obesity and the Uterine Immune Cell Landscape: The Shaping Role of Inflammation. Int J Mol Sci (2020) 21:11. doi: 10.3390/ijms21113776 PMC7312391 32471078

[72] Barber EM Pollard JW. The Uterine NK Cell Population Requires IL-15 But These Cells are Not Required for Pregnancy Nor the Resolution of a *Listeria Monocytogenes* Infection. J Immunol (2003) 171:1. doi: 10.4049/jimmunol.171.1.37 12816981

[73] Zenclussen ML Casalis PA El-Mousleh T Rebelo S Langwisch S Linzke N. Haem Oxygenase-1 Dictates Intrauterine Fetal Survival in Mice *via* Carbon Monoxide. J Pathol (2011) 225:2. doi: 10.1002/path.2946 21744344

[74] Fisher AL Sangkhae V Balušíková K Palaskas NJ Ganz T Nemeth E. Iron-Dependent Apoptosis Causes Embryotoxicity in Inflamed and Obese Pregnancy. Nat Commun (2021) 12:1. doi: 10.1038/s41467-021-24333-z 34188052 PMC8242011

[75] Kaislasuo J Simpson S Petersen JF Peng G Aldo P Lokkegaard E. IL-10 to Tnfα Ratios Throughout Early First Trimester can Discriminate Healthy Pregnancies From Pregnancy Losses. Am J Reprod Immunol 83:1. doi: 10.1111/aji.13195 PMC8295882 31585488

[76] Latchoumycandane C Marathe GK Zhang R McIntyre TM. Oxidatively Truncated Phospholipids are Required Agents of Tumor Necrosis Factor α (Tnfα)-Induced Apoptosis. J Biol Chem (2012) 287:21. doi: 10.1074/jbc.M111.300012 22433871 PMC3366783

[77] Li C Deng X Xie X Liu Y Friedmann Angeli JP Lai L. Activation of Glutathione Peroxidase 4 as a Novel Anti-Inflammatory Strategy. Front Pharmacol (2018) 9:1120. doi: 10.3389/fphar.2018.01120 30337875 PMC6178849

[78] Kwon MY Park E Lee SJ Chung SW. Heme Oxygenase-1 Accelerates Erastin-Induced Ferroptotic Cell Death. Oncotarget (2015) 6:27. doi: 10.18632/oncotarget.5162 PMC4695193 26405158

[79] Sharma L Kaur J Shukla G. Role of Oxidative Stress and Apoptosis in the Placental Pathology of *Plasmodium Berghei* Infected Mice. PloS One (2012) 7:3. doi: 10.1371/journal.pone.0032694 PMC3291651 22396790

[80] Akanbi OM Odaibo AB Ademowo OG. Effect of Antimalarial Drugs and Malaria Infection on Oxidative Stress in Pregnant Women. Afr J Reprod Health (2010) 14:3. 21495615

[81] Imai H Hirao F Sakamoto T Sekine K Mizukura Y Saito M. Early Embryonic Lethality Caused by Targeted Disruption of the Mouse PHGPx Gene. Biochem Biophys Res Commun (2003) 305:2. doi: 10.1016/s0006-291x(03)00734-4 12745070

[82] Yant LJ Ran Q Rao L Van Remmen H Shibatani T Belter JG. The Selenoprotein GPX4 is Essential for Mouse Development and Protects From Radiation and Oxidative Damage Insults. Free Radic Biol Med (2003) 34:4. doi: 10.1016/s0891-5849(02)01360-6 12566075

[83] Campbell NK Fitzgerald HK Dunne A. Regulation of Inflammation by the Antioxidant Haem Oxygenase 1. Nat Rev Immunol (2021) 21:7. doi: 10.1038/s41577-020-00491-x 33514947

[84] Almeida MPO Mota CM Mineo TWP Ferro EAV Barbosa BF Silva NM. Heme Oxygenase-1 Induction in Human BeWo Trophoblast Cells Decreases *Toxoplasma Gondii* Proliferation in Association With the Upregulation of P38 MAPK Phosphorylation and IL-6 Production. Front Microbiol (2021) 12:659028. doi: 10.3389/fmicb.2021.659028 33912151 PMC8071940

[85] Suttner DM Dennery PA. Reversal of HO-1 Related Cytoprotection With Increased Expression is Due to Reactive Iron. FASEB J (1999) 13:13. doi: 10.1096/fasebj.13.13.1800 10506583

[86] Wang W Sung N Gilman-Sachs A Kwak-Kim J. T Helper (Th) Cell Profiles in Pregnancy and Recurrent Pregnancy Losses: Th1/Th2/Th9/Th17/Th22/Tfh Cells. Front Immunol (2020) 11:2025. doi: 10.3389/fimmu.2020.02025 32973809 PMC7461801

[87] Terry CM Clikeman JA Hoidal JR Callahan KS. Effect of Tumor Necrosis Factor-Alpha and Interleukin-1 Alpha on Heme Oxygenase-1 Expression in Human Endothelial Cells. Am J Physiol (1998) 274:H883–91. doi: 10.1152/ajpheart.1998.274.3.H883 9530200

[88] Mu J Slevin JC Qu D McCormick S Adamson SL. *In Vivo* Quantification of Embryonic and Placental Growth During Gestation in Mice Using Micro-Ultrasound. Reprod Biol Endocrinol (2008) 6:34. doi: 10.1186/1477-7827-6-34 18700008 PMC2527569

[89] Tseng AM Mahnke AH Wells AB Salem NA Allan AM Roberts VH. Maternal Circulating miRNAs That Predict Infant FASD Outcomes Influence Placental Maturation. Life Sci Alliance (2019) 2:e201800252. doi: 10.26508/lsa.201800252 30833415 PMC6399548

[90] Bankhead P Loughrey MB Fernández JA Dombrowski Y McArt DG Dunne PD. QuPath: Open Source Software for Digital Pathology Image Analysis. Sci Rep (2017) 7:1. doi: 10.1038/s41598-017-17204-5 29203879 PMC5715110

[91] Schindelin J Arganda-Carreras I Frise E Kaynig V Longair M Pietzsch T. Fiji: An Open-Source Platform for Biological-Image Analysis. Nat Methods (2012) 9:7. doi: 10.1038/nmeth.2019 PMC3855844 22743772

[92] Paffaro VA Jr. Bizinotto MC Joazeiro PP Yamada AT. Subset Classification of Mouse Uterine Natural Killer Cells by DBA Lectin Reactivity. Placenta (2003) 24:5. doi: 10.1053/plac.2002.0919 12744924

[93] Bradford MM. A Rapid and Sensitive Method for the Quantitation of Microgram Quantities of Protein Utilizing the Principle of Protein-Dye Binding. Anal Biochem (1976) 72:1–2. doi: 10.1016/0003-2697(76)90527-3 942051

[94] Huy NT Xuan Trang DT Uyen DT Sasai M Harada S Kamei K. An Improved Colorimetric Method for Quantitation of Heme Using Tetramethylbenzidine as Substrate. Anal Biochem (2005) 344:2. doi: 10.1016/j.ab.2005.06.022 16091279

[95] Lima Júnior JP Franco RR Saraiva AL Moraes IB Espindola FS. *Anacardium Humile* St. Hil as a Novel Source of Antioxidant, Antiglycation and α-Amylase Inhibitors Molecules With Potential for Management of Oxidative Stress and Diabetes. J Ethnopharmacol (2021) 268:113667. doi: 10.1016/j.jep.2020.113667 33301920

[96] Livak KJ Schmittgen TD. Analysis of Relative Gene Expression Data Using Real-Time Quantitative PCR and the 2(-Delta Delta C(T)) Method. Methods (2001) 25:4. doi: 10.1006/meth.2001.1262 11846609

