# Supplementary material for: Inhibition of Heme Oxygenase-1 by Zinc Protoporphyrin IX Improves Adverse Pregnancy Outcomes in Malaria During Early Gestation
Source: Front Immunol. 2022 May 10;13:879158. doi: 10.3389/fimmu.2022.879158 (PMC9127164; doi:10.3389/fimmu.2022.879158)
Supplement: Supplementary file 1 [file DataSheet_1.pdf]

Supplementary Material

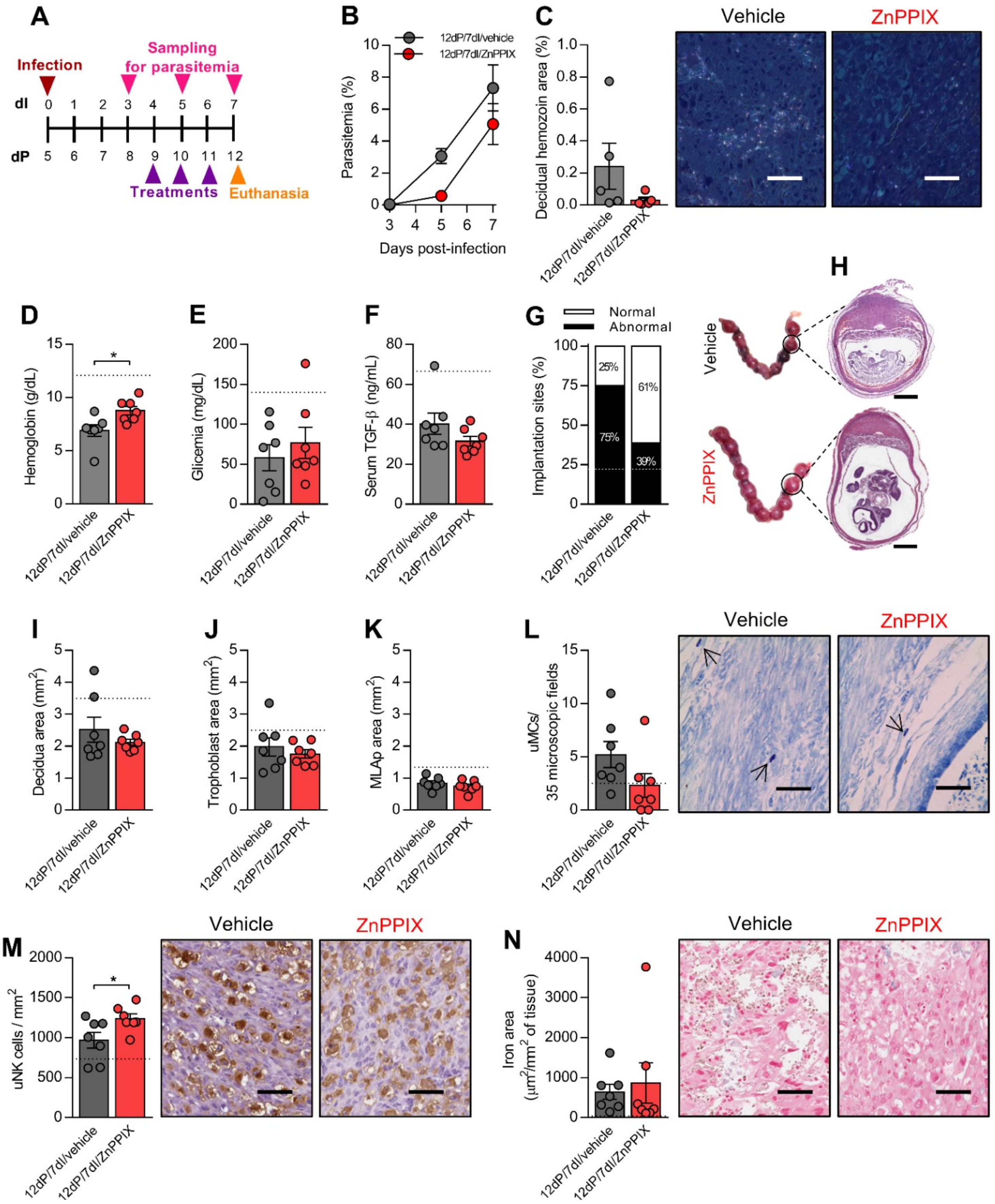

Supplementary Figure S1. Inhibition of HO-1 does not significantly ameliorate alterations induced by malaria infection in mid-pregnancy. (A) An experimental design for the pharmacological inhibition of HO-1 by ZnPPIX during malaria in mid-gestation was performed. **(B)** Parasitemia levels were estimated by flow cytometry. Each symbol represents the mean of all animals within the indicated group ± SEM (n = 7 mice/group). **(C)** Hemozoin was detected in decidual tissue using polarized light microscopy, scale bar 50 µm. **(D-F)** Hemoglobin, blood glucose and serum TGF-β levels were measured. **(G-H)** The implantations sites were classified as normal or abnormal **(G)** according to macroscopic aspect (n = 7 mice/group) **(H)**, histologically processed and stained by H&E **(H)**, scale bar 50 µm. **(I-K)** Decidua **(I)**, trophoblast **(J)** and MLAp **(K)** areas were delimited and quantified. **(L)** uMCs were detected and quantified in Toluidine Blue stained implantation sites, scale bar 50 µm. **(M)** uNK cells were detected and quantified in the decidua of DBA lectin-stained implantation sites, scale bar 50 µm. **(N)** Quantification of iron deposition and representative images of iron-stained tissue, scale bar 50 µm. Unless otherwise stated, each symbol represents the measure of one mouse and results represent mean ± SEM. The dashed lines indicate the mean results of pregnant uninfected and untreated mice for each parameter evaluated. Data was analyzed by Two-way ANOVA followed by Bonferroni's post-hoc comparisons tests **(B)**, unpaired t-test **(C-F,I-N)** and Mann-Whitney test **(G)**. NP: non-pregnant, NI: non-infected, dP: days of pregnancy, dI: days of infection. *: $p < 0.05$.

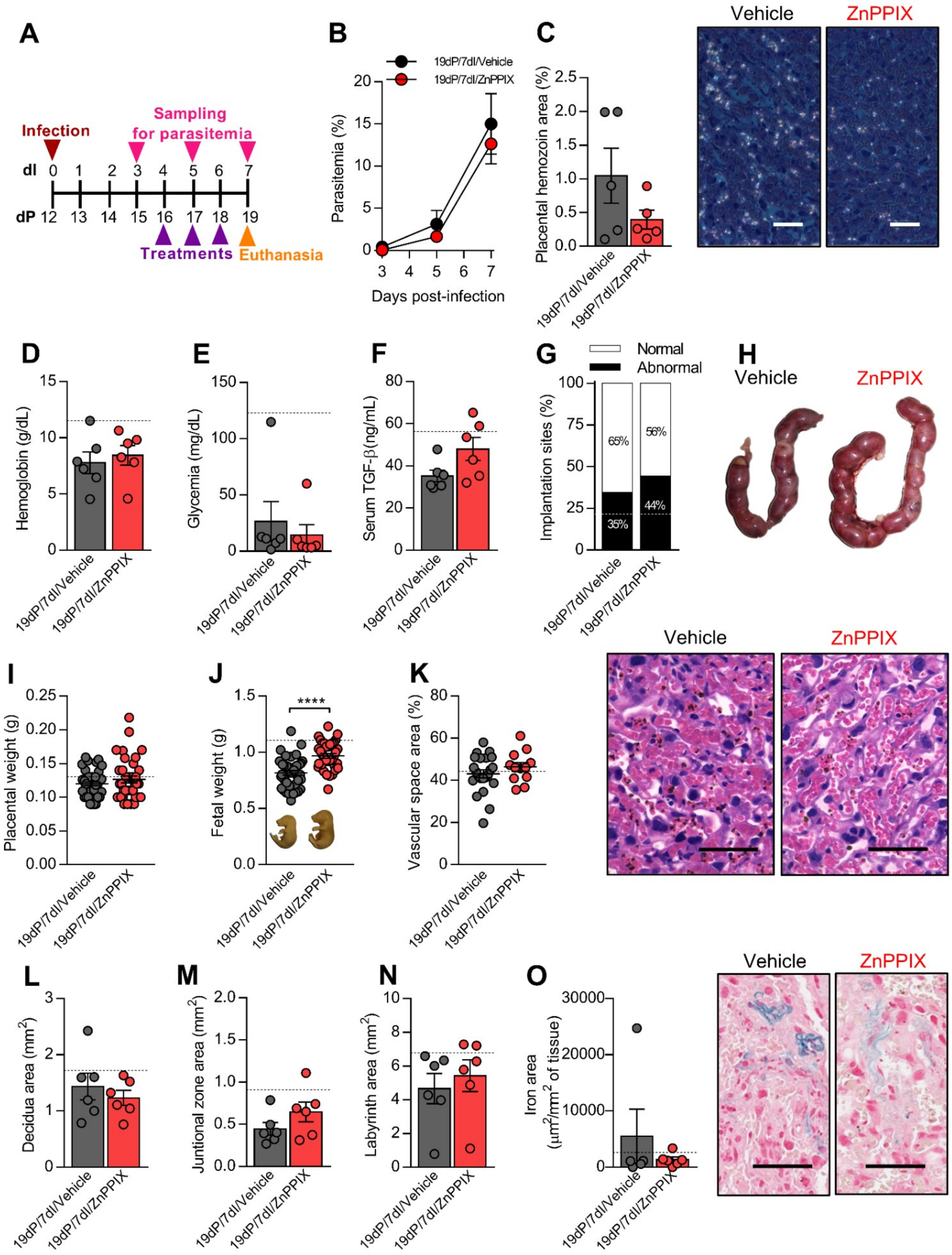

A
Infection
Sampling for parasitemia
dI 0 1 2 3 4 5 6 7
dP 12 13 14 15 16 17 18 19
Treatments
Euthanasia
B
19dP/7dI/Vehicle
19dP/7dI/ZnPPIX
Parasitemia (%)
Days post-infection
C
Placental hemozoin area (%)
Vehicle
ZnPPIX
D
Hemoglobin (g/dL)
E
Glycemia (mg/dL)
F
Serum TGF-β (ng/mL)
G
Normal
Abnormal
Implantation sites (%)
65%
56%
35%
44%
H
Vehicle
ZnPPIX
I
Placental weight (g)
J
Fetal weight (g)
****
K
Vascular space area (%)
Vehicle
ZnPPIX
L
Decidua area (mm²)
M
Juntional zone area (mm²)
N
Labyrinth area (mm²)
O
Iron area (μm²/mm² of tissue)
Vehicle
ZnPPIX

Supplementary Figure S2. Inhibition of HO-1 does not significantly ameliorate alterations induced by malaria infection in late pregnancy. **(A)** An experimental design for the pharmacological inhibition of HO-1 by ZnPPIX during malaria in late gestation was performed. **(B)** Parasitemia levels were estimated by flow cytometry. Each symbol represents the mean of all animals within the indicated group ± SEM (n = 6 mice/group). **(C)** Hemozoin was detected in decidual tissue using polarized light microscopy, scale bar 50 µm. **(D-F)** Hemoglobin, blood glucose and serum TGF-β levels were measured. **(G-H)** The implantations sites were classified as normal or abnormal **(G)** according to macroscopic aspect **(H)** (n = 6 mice/group). **(I-J)** Fetal and placental weight were recorded. Each point represents the measurement of a placenta or fetus derived from 6 litters per group. **(K)** Vascular space area in labyrinth layer was estimated in placenta sections stained with H&E, scale bar 50 µm. **(L-N)** Placental layers decidua **(L)**, junctional zone **(M)** and labyrinth **(N)** were delimited and size measured. **(O)** Quantification of iron deposition and representative images of iron-stained tissue, scale bar 50 µm. Unless otherwise stated, each symbol represents the measure of one mouse and results represent mean ± SEM. The dashed lines indicate the mean results of pregnant uninfected and untreated mice for each parameter evaluated. Data was analyzed by Two-way ANOVA followed by Bonferroni's post-hoc comparisons tests **(B)**, unpaired t-test **(C,D,F,G,J-M,O)** and Mann-Whitney test **(E,I,N)**. NP: non-pregnant, NI: non-infected, dP: days of pregnancy, dI: days of infection. ****: $p < 0.0001$.

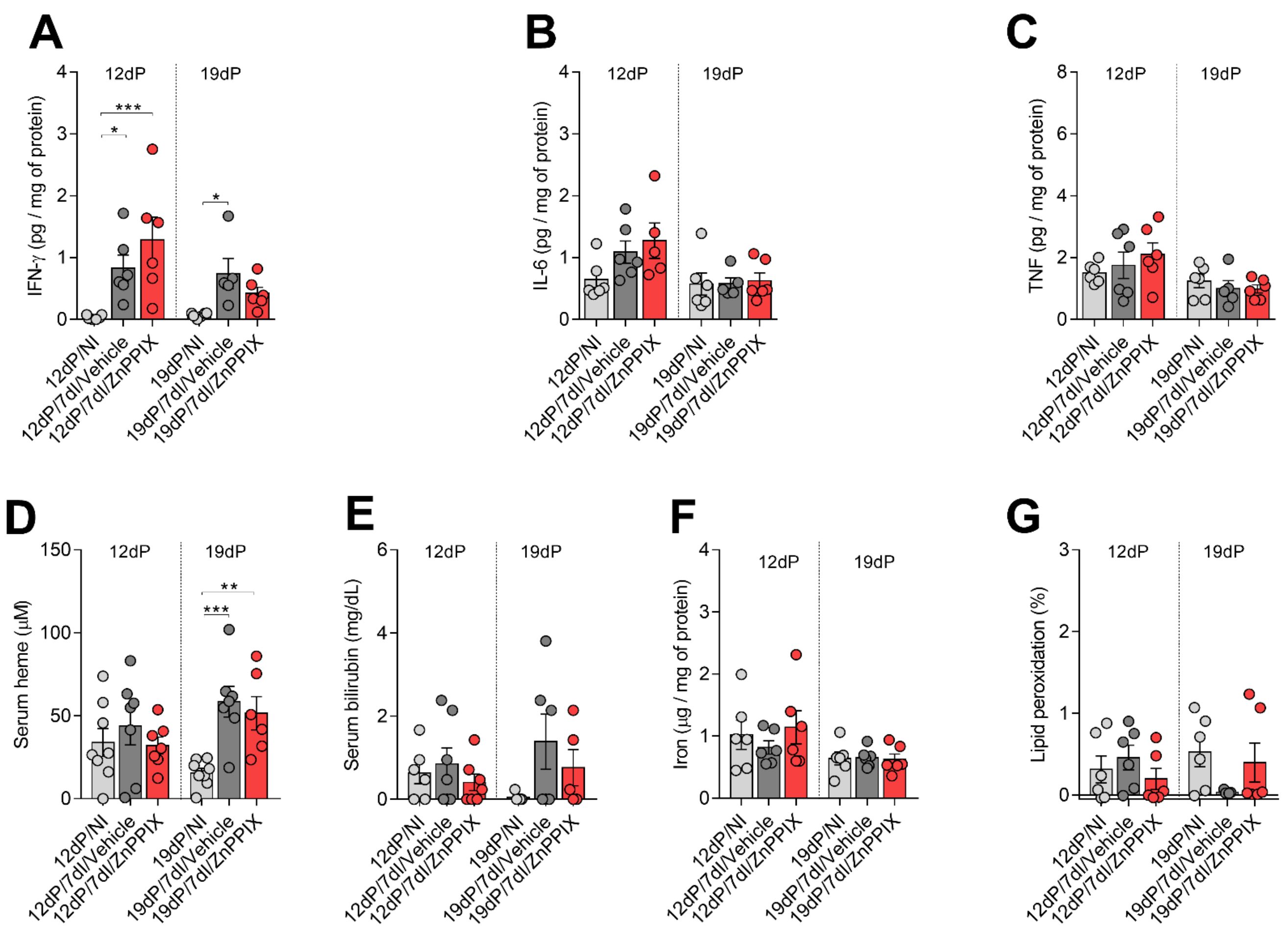

Supplementary Figure S3. HO-1 inhibitory treatment does not affect oxidative stress and inflammation during malaria in mid and late gestation. (A-C) Levels of the cytokines IFN-γ **(A)**, IL-6 **(B)** and TNF **(C)** were measured in samples of uterus/placenta of indicated groups. **(D-E)** Serum levels of heme and bilirubin were measured. **(F-G)** Iron concentration and lipid peroxidation levels were quantified in tissue samples from implantation sites. Each symbol represents the measure of one mouse and results represent mean ± SEM. Data was analyzed by One-way ANOVA with Bonferroni's post-hoc comparisons tests **(A,C,D,F)** or Kruskal-Wallis test with Dunn's post-hoc comparisons tests **(B,E,G)**. NP: non-pregnant, NI: non-infected, dP: days of pregnancy, dI: days of infection. *: $p < 0.05$; **: $p < 0.01$; ***: $p < 0.001$.

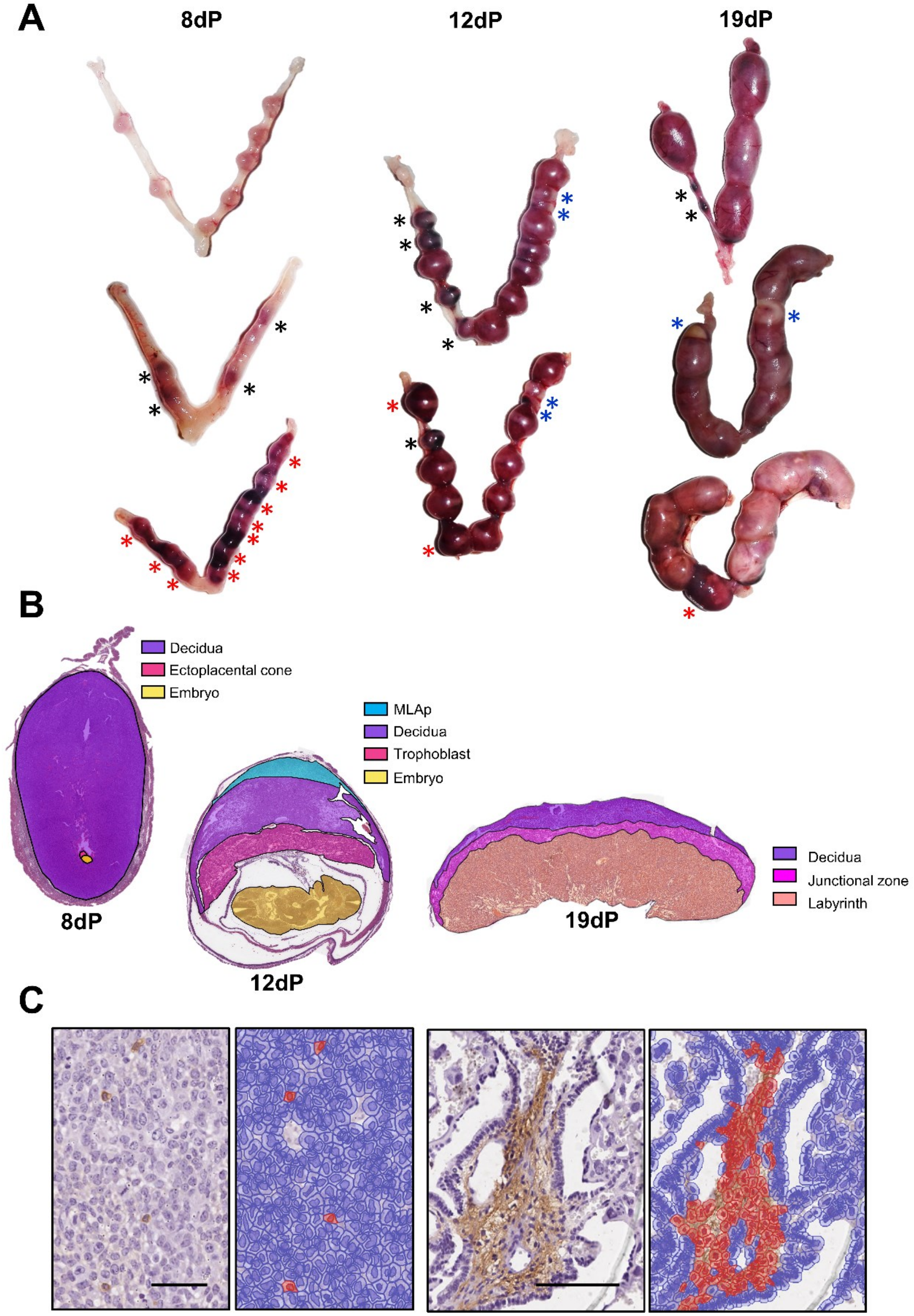
A
8dP
12dP
19dP
B
Decidua
Ectoplacental cone
Embryo
MLAp
Decidua
Trophoblast
Embryo
8dP
12dP
19dP
Decidua
Junctional zone
Labyrinth
C

Supplementary Figure S4. Representative images of macro and microscopic analysis of uteroplacental tissue. (A) Representative images of implantation sites in early, mid and late gestation. Implantation sites were considered abnormal when they presented necrotic aspect (black asterisks), hemorrhagic (red asterisks), paleness and/or smaller size (blue asterisks). **(B)** Scheme for delimitation of uteroplacental layers in early, mid and late gestation. **(C)** Representative images of the automated analysis for detection of HO-1^+ cells. DAB-stained cells are highlighted in red. Left panel: decidua, scale bar 50 µm; right panel: chorionic plate, scale bar 100 µm. dP: days of pregnancy.

Table S1. Fetal biometry measurements in fetuses of BALB/c mice infected with *P. berghei* ANKA and treated or not with ZnPPIX

Parameter	19dP/7dI/vehicle	19dP/7dI/ZnPPIX	p-value
CRL (cm)			
Mean ± SEM	1.936 ± 0.0250	2.159 ± 0.0221	**<0.0001**
Minimum	1.516	1.838	
Maximum	2.353	2.483	
SOD (cm)			
Mean ± SEM	0.999 ± 0.0090	1.118 ± 0.0100	**<0.0001**
Minimum	0.848	0.950	
Maximum	1.109	1.281	
APD (cm)			
Mean ± SEM	0.816 ± 0.0104	0.887 ± 0.0059	**<0.0001**
Minimum	0.587	0.802	
Maximum	1.054	0.982	
BPD (cm)			
Mean ± SEM	0.696 ± 0.0074	0.776 ± 0.0060	**<0.0001**
Minimum	0.557	0.692	
Maximum	0.795	0.873	

CRL: Crown-rump length, SOD: snout-occipital distance, APD: abdominal anteroposterior diameter, BPD: biparietal diameter, SEM: standard error of the mean. 19dP/7dI/vehicle group (n=6 litters, 54 pups); 19dP/7dI/ZnPPIX group (n=6 litters, 40 pups). The differences between the groups were determined by unpaired t-test . p-values <0.05 were considered significant.
